# Transcription factor Creb3l1 maintains proteostasis in neuroendocrine cells

**DOI:** 10.1016/j.molmet.2022.101542

**Published:** 2022-07-06

**Authors:** Mingkwan Greenwood, Benjamin T. Gillard, Rizwan Farrukh, Alex Paterson, Ferdinand Althammer, Valery Grinevich, David Murphy, Michael P. Greenwood

**Affiliations:** 1 Molecular Neuroendocrinology Research Group, Bristol Medical School: Translational Health Sciences, University of Bristol, Dorothy Hodgkin Building, Bristol, United Kingdom; 2 Institute of Human Genetics, University Hospital Heidelberg, Heidelberg, Germany; 3 Department of Neuropeptide Research in Psychiatry, Central Institute of Mental Health, Medical Faculty Mannheim, University of Heidelberg, Mannheim, Germany; 4 Center for Neuroinflammation and Cardiometabolic Diseases, Georgia State University, Atlanta, GA, USA

**Keywords:** Unfolded protein response, Endoplasmic reticulum stress, Ribosome, Neuropeptide, Hypothalamus, PERK, WD, water deprivation, MCN, magnocellular neurone

## Abstract

**Objectives:**

Dynamic changes to neuropeptide hormone synthesis and secretion by hypothalamic neuroendocrine cells is essential to ensure metabolic homeostasis. The specialised molecular mechanisms that allow neuroendocrine cells to synthesise and secrete vast quantities of neuropeptides remain ill defined. The objective of this study was to identify novel genes and pathways controlled by transcription factor and endoplasmic reticulum stress sensor Creb3l1 which is robustly activated in hypothalamic magnocellular neurones in response to increased demand for protein synthesis.

**Methods:**

We adopted a multiomic strategy to investigate specific roles of Creb3l1 in rat magnocellular neurones. We first performed chromatin immunoprecipitation followed by genome sequencing (ChIP-seq) to identify Creb3l1 genomic targets and then integrated this data with RNA sequencing data from physiologically stimulated and Creb3l1 knockdown magnocellular neurones.

**Results:**

The data converged on Creb3l1 targets that code for ribosomal proteins and endoplasmic reticulum proteins crucial for the maintenance of cellular proteostasis. We validated genes that compose the PERK arm of the unfolded protein response pathway including *Eif2ak3*, *Eif2s1*, *Atf4* and *Ddit3* as direct Creb3l1 targets. Importantly, knockdown of Creb3l1 in the hypothalamus led to a dramatic depletion in neuropeptide synthesis and secretion. The physiological outcomes from studies of paraventricular and supraoptic nuclei Creb3l1 knockdown animals were changes to food and water consumption.

**Conclusion:**

Collectively, our data identify Creb3l1 as a comprehensive controller of the PERK signalling pathway in magnocellular neurones in response to physiological stimulation. The broad regulation of neuropeptide synthesis and secretion by Creb3l1 presents a new therapeutic strategy for metabolic diseases.

## Highlights

• A multiomic investigation of transcription factor Creb3l1 in the hypothalamus.
• Creb3l1 targets ER and ribosomal genes to preserve cellular proteostasis.
• Identification of Creb3l1 as a major controller of the PERK arm of the UPR pathway.
• Knockdown of Creb3l1 in the PVN and SON inhibits hormone synthesis and secretion.
• Hypothalamic Creb3l1 expression regulates food and water intake.

## 1 Introduction

Specialised cells exist in tissues throughout the body that possess the unique capacity to synthesise and secrete vast quantities of protein to match physiological demands. This places enormous pressure on the secretory pathway in these cells. The endoplasmic reticulum (ER) is the gateway to this pathway and is crucially responsible for the folding of proteins [1]. To monitor protein folding capacity as a result of changing secretory requirements, cells utilise intracellular signalling pathways to ensure that protein folding capacity remains in balance with protein folding demand. When this threshold is exceeded, the accumulation of misfolded protein triggers cellular stress responses that sets into motion a signalling pathway known as the unfolded protein response (UPR) to protect cells and restores proteostasis [2].

The UPR signalling network helps to recover ER function through the activation of chaperone proteins such as immunoglobulin heavy chain binding protein (BIP) to facilitate folding and assembly of nascent polypeptides, inhibit general protein translation to reduce workload in the ER, and increase degradation of misfolded proteins through a process called ER-associated degradation (ERAD) [3]. The ER stress response and UPR is driven by at least three major ER stress sensors: protein kinase RNA-like ER kinase (PERK), inositol-requiring protein 1α (IRE1α), and activating transcription factor (TF) 6 (ATF6) [1]. The PERK phosphorylates eukaryotic initiation factor eIF2α disrupting the assembly of the ribosome, thus directly regulating translation initiation to reduce general protein synthesis. This results in a shift in translation to favour eIF2α downstream effectors activating TF 4 (ATF4) and C/EBP-homologous protein (CHOP) to adapt to ER stress [4]. Activation of IRE1α results in the splicing of the X box binding protein 1u (*Xbp1u*) RNA to generate the *Xbp1s* transcript encoding the XBP1 TF that is responsible for promoting the expression genes involved in protein folding and ERAD [5]. Activation of ATF6, a basic leucine zipper (bZIP) TF, controls the transcription of genes involved in ERAD and BIP and XBP1 [6].

Despite the robustness of the UPR pathway, cells often work near their limits of secretory capacity in physiological situations. Disturbances in the UPR have been implicated in several diseases including metabolic disease, neurodegenerative diseases, and cancer [7]. The sustained activation of the UPR is linked to apoptosis in many cells types, but cancer cells are known to exploit this pathway to promote proliferation and metastasis [8]. In neurones, constitutive activity of the UPR is required, which leads to increased sensitivity of the brain to abnormalities in UPR function [9]. The duration of activation can lead to unsustainable demands on the protein-folding machinery, causing chronic ER stress. The accumulation of misfolded and aggregated proteins is reflected in studies of neurodegenerative diseases like Parkinson's disease (PD) and Alzheimer's disease (AD) where the UPR is not sufficient to recover cellular proteolysis and becomes a threat to cell survival [10]. Therefore, investigation the UPR pathway in specialised secretory cells that can handle large protein loads may offer insight into treatments for these diseases.

To understand how neuronal cells cope with dynamically changing protein loads we have studied specialised neuroendocrine cells in the hypothalamus. The magnocellular neurosecretory cells (MCNs) in the paraventricular nucleus (PVN) and supraoptic nucleus (SON) of the hypothalamus are exemplars of neuroendocrine cells in the brain synthesising vast quantities of neuropeptides for release centrally from dendrites and collateral fibres and peripherally via nerve terminals in the neurohypophysis [11]. These specialised neuronal cells are constantly being challenged by altered physiological demands and in some cases pathological perturbations. When demand increases, for example during water deprivation (WD) [12], a plethora of ultrastructural changes ensue including increased MCN cell size, expanded organelles, and an increase in the number of nucleoli, all to support demands for protein synthesis [13,14]. These changes have also been observed in ageing rodents and humans [14]. Interestingly, MCNs appear resistant to many forms of neurodegenerative pathology compared to other hypothalamic and brain regions suggesting that MCNs possess specialised UPR regulatory mechanisms [15]. Knowledge of these mechanisms may provide insight into alleviating disruptions to UPR function in neurodegenerative diseases.

When stimulated, we have shown that MCNs exhibit “physiological ER stress” which leads to increased expression of ER stress sensors [16]. We were the first to describe expression of ER stress sensor cAMP responsive element-binding protein 3 like 1 (Creb3l1) in MCNs of the hypothalamus [17]. We have gone on to characterise its mechanism of activation and functions in MCNs in the regulation of vasopressin (AVP) and oxytocin (OXT) synthesis in response to increased physiological demand [[16], [17], [18], [19], [20], [21]]. Creb3l1 is a TF of the CREB/ATF family that, like ATF6, is activated by regulated intramembrane proteolysis (RIP) [22]. The expression of Creb3l1 has become synonymous with specialised secretory cell-types throughout the body, particularly cells with high demand for protein synthesis and secretion [17,[23], [24], [25]]. The first evidence to support Creb3l1 in cell secretion was from Creb3l1 knockout mice that exhibit severe osteopenia due to reduced synthesis and secretion of bone matrix proteins [26]. Like other ER stress sensors, Creb3l1 has been shown to control the transcription of essential UPR components BIP [27] and XBP1 [16]. In the SON, we identified genes *Ddit3* that codes for the CHOP protein and *Xbp1* as transcriptional targets for Creb3l1 [16].

To determine the entirety of the Creb3l1 regulome in MCNs in response to physiological stress, we used chromatin immunoprecipitation followed by genome sequencing (ChIP-seq) to map global DNA binding sites. Our approach has been to integrate ChIP-seq data with RNA-seq transcriptome datasets from physiological stimulation and viral manipulation of Creb3l1 expression. This has provided an integrated signalling-gene regulation network for this TF illuminating changes to cell pathways and function, an approach that has led us to understand the physiological changes that occur in MCNs to cope with excessive protein demands.

## 2 Materials and methods

### 2.1 Animals

All experiments were performed under UK Home Office licence PP9294977 held under, and in strict accordance with, the provisions of the UK Animals (Scientific Procedures) Act (1986); they had also been approved by the University of Bristol Animal Welfare and Ethical Review Board. Animal sample sizes were calculated by making an estimate of variability from previous experiments that we have performed with two groups, using similar approaches, under similar conditions, in rats. These data provide an estimate of the expected standard deviation of the primary outcome and then power calculations were used to calculate the sample size for the experimental group [28]. In some cases, the same animal was able to serve as its own control with control virus injected into one SON and experimental virus into the other (referred to as SONs per group). All studies were performed with time-matched experimental controls run in parallel and sampled on the same day. For animal studies with two groups, randomisation was performed by the flip of a coin to determine which group the animal was assigned. Viral injections for each animal study were completed in 1–2 days. In our previous studies injections were missed in approximately 10% of animals so numbers were accordingly increased.

Male Sprague Dawley rats weighing 250–274 g were purchased from commercial supplier Envigo (RRID: 70508). Rats were housed under a 12:12 light/dark cycle at a temperature of 21–22 °C and a relative humidity of 40–50% with food and water *ad libitum* unless stated. Through the University of Bristol Animal Management Information System, animals are randomly assigned to cages by Animal Services Unit staff. The number of cages, and the number of animals per cage, is pre-set by the investigator on AMIS, but the allocation is independently performed. Rats were housed in groups of 3–4 for a 1–2-week period of acclimation before experimentation. After surgical procedures animals were singly housed for at least 7 days. If food and water intake measures were not required from viral injected animals, they were returned to their original groups 7 days post-surgery. Cages contained sawdust, bedding material, cardboard tubes, and wooden chews for enrichment. In some experimental series rats were placed in metabolic cages (Techniplast) to allow for precise daily measures of food and water intake alongside urine output. Plastic chew toys (discs) were suspended from the cage lid and actively gnawed providing enrichment to their environment. Animals were first acclimatised to metabolic cages for 2 days. Measures of food, water, and urine were performed by weight at the start of the light phase. For WD studies, drinking water was removed at the middle of the light phase.

### 2.2 Plasma and hormone measures

Rats were humanely killed by striking the cranium and then immediately decapitated with a small animal guillotine (Harvard Apparatus). Trunk blood was collected in ethylenediamine tetraacetic acid (EDTA)-coated tubes (1 mg/ml of blood) and centrifuged at 1,600×*g* for 20 min at 4 °C. Plasma for hormone measures was collected in 1 ml aliquots and snap frozen in liquid nitrogen before storage at −80 °C. Brains were rapidly removed and covered with powdered dry ice. Pituitaries were placed in 1.5 ml tubes containing 0.5 ml of 0.1 M HCl and stored at −80 °C. Plasma and urine osmolality measures were performed by freezing point depression using a Roebling micro-osmometer (Camlab). Plasma hormone measures: Plasma concentrations of copeptin and OXT were determined in extracted samples by ELISA (Copeptin, Develop, DL-CPP-Ra; OXT, Enzo Life Sciences, ADI-901-153 A, RRID:AB_2,815,012). The extraction method was reverse phase C18 columns (Phenomenex) as described by Enzo Life Sciences and samples were dried under a gentle flow of nitrogen gas. Samples were reconstituted by vortexing in assay buffer and assayed immediately in duplicate in accordance with manufacturer's protocols. Pituitary hormone measures were performed as described previously [12] using AVP^8^-Vasopressin ELISA (Enzo Life Sciences, ADI-900-017 A) and OXT-Oxytocin ELISA kits (Enzo Life Sciences, ADI-901-153 A, RRID:AB_2,815,012).

### 2.3 Cells and treatments

Mouse pituitary cell line AtT20/D16v-F2 (Sigma–Aldrich, 94050406, RRID: CVCL_4109), Human Embryonic Kidney cells HEK293T/17 (ATTC, CRL-11268, RRID: CVCL_1926), Neuro 2a cells N2a (ATTC, CCL-131, RRID: CVCL_0470), breast cancer cell line MCF7 (a kind gift from Dr. Stephen Lolait, University of Bristol), and hypothalamic 4 B cells (a kind gift from Dr. John Kasckow [29]) were cultured in DMEM (Sigma, D6546) supplemented with 10% (v/v) heat-inactivated foetal bovine serum (Sigma–Aldrich; F9665), 2 mM l-glutamine (Gibco, 25030) and 100 unit/ml of penicillin-streptomycin (Gibco, 15140). Cells were incubated at 37 °C in a humidified incubator with 5% (v/v) CO_2_. Cells were seeded onto tissue culture plates to 60%–70% confluence for experiments.

### 2.4 Chromatin immunoprecipitation sequencing

We performed ChIP using SimpleChIP Plus Enzymatic Chromatin IP Kit (Cell Signaling Technology, 38191). WD rats (n = 9) were killed by striking of the cranium and brains were rapidly removed and placed into a rodent brain matrix (ASI Instruments, RBM-4000C) chilled on ice. A 2 mm coronal brain slice excised between two razor blades and placed onto a 10 cm upturned petri dish chilled on ice. A 2 mm in diameter sample corer (Fine Science Tools, 18035–02) was used to collect pairs of SONs and a single punch to collect the PVN using the third ventricle and fornix as anatomical references. Tissue samples were expelled from the sample corer into 1.5 ml Biomasher tubes (Takara Bio, Cat No. 9791 A) containing 200 μl of ice-cold phosphate buffered saline (PBS) and 1 x protease inhibitor cocktail (PBS+). Samples were disrupted for 15 s with a Biomasher homogeniser, and the volume was increased to 500 μl with PBS+. To crosslink proteins to the DNA, 22.5 μl of 37% formaldehyde (Sigma–Aldrich, F8775) was added to each sample followed by end over end mixing (Tube Rotor, VWR, 444–0502) for 20 min at room temperature (RT). Crosslinking was stopped by adding 50 μl of 10x glycine solution followed by end over end mixing for 5 min at RT. Tissue samples were pelleted by centrifugation at 500×*g* for 5 min at RT. The supernatant was removed, and pellets washed with 500 μl of PBS+. This step was repeated, and pellets were frozen on dry ice and stored at −80 °C. Pellets were thawed on ice and 100 μl of ice-cold PBS+ was added to each pellet. Sample disaggregation was performed by 20 strokes with a Biomasher homogeniser and samples from 9 animals were pooled. Samples were centrifuged at 2000×*g* at 4 °C using a swing out rotor. The pellet was resuspended in 5 ml of buffer A containing dithiothreitol (DTT) and protease inhibitors and incubated on ice for 10 min, inverting every 3 min. Nuclei were pelleted by centrifugation at 2000×*g* for 5 min at 4 °C. After removal of supernatant the pellet was resuspended in 5 ml of buffer B containing DTT and centrifuged at 2000×*g* for 5 min at 4 °C. The supernatant was removed, and pellet was resuspended in 500 μl of buffer B. To this sample 3.75 μl of Micrococcal Nuclease (Cell Signaling Technology, 10011) was added and incubated at 37 °C for 20 min in a water bath to digest the DNA, mixing by inversion every 3 min. The digestion was stopped by the addition of 50 μl of 0.5 M EDTA and cooling on ice for 2 min. Nuclei were pelleted by centrifugation at 16,000×*g* for 1 min at 4 °C and the pellet was resuspended in 500 μl of 1x ChIP buffer containing protease inhibitors and incubated on ice for 10 min. The lysate was sonicated for 3 sets of 20 s pulses using a MSE Soniprep 150 with 30 s incubation on ice between each pulse. The lysates were clarified by centrifugation at 10,000×*g* for 10 min at 4 °C. We started ChIP using 7.3 μg of digested crosslinked chromatin and followed the manufacturers protocol. We used 2 μg of previously validated (ENCODE project ENCSR109YGM) Creb3l1 ChIP antibody (Sigma–Aldrich, HPA024069, RRID: AB_1854750) for immunoprecipitation (IP) and incubated with the chromatin overnight at 4 °C with end over end mixing. A 10 μl aliquot of diluted chromatin (input) was stored at −20 °C. The following day ChIP-Grade protein G magnetic beads were added to IP samples and incubated for 2 h at 4 °C with end over end mixing. The beads were captured by magnetic separation and washed with low and high salt washes. The chromatin was eluted from the antibody/magnetic beads at 65 °C and a speed of 1200 rpm using a HM100-Pro Digital Thermo Mixer. The cross-links for the input sample and IP sample were reversed by adding 5 M NaCl and RNase A for 30 min at 37 °C followed by the addition of proteinase K and incubation at 65 °C for 2 h. DNA was purified using Zymo Research ChIP DNA Clean and Concentrator kit (D5205) and eluted in 12 μl. The total amount of DNA recovered as quantified by a Qubit 4 Fluorometer was 1.56 ng of ChIP DNA.

We used the ChIP-seq service provided by Zymo Research for genome-wide mapping of protein to DNA interactions and bioinformatic analysis. The sheared DNA ranged from 100 to 700 bp as determined by Agilent TapeStation 2200. The ChIP DNA libraries were prepared from 1 ng of ChIP DNA and 1 ng of Input DNA using a proprietary library protocol from Zymo Research. Chip-seq libraries were quantified by Qubit and diluted to 2 nM before being multiplexed and run on the Next-Generation Sequencing platform Hiseq. The input Chip-seq sample received more than 56.1 million reads and the ChIP DNA sample 57.8 million reads with >94% mapping ratio to rat genome rn6. The peak call was performed with MACS version 2.1.0.20150731 by Zymo Research. ChIP-seq traces were visualised in Integrative Genomics Viewer (IGV) 2.11.0 [30].

### 2.5 De novo Creb3l1 motif discovery

We used motif-based sequence analysis tools in MEME suite version 5.4.1 for *de novo* motif discovery and investigation. We performed discriminative motif discovery of ungapped motifs using Sensitive, Through, Rapid, Enriched Motif Elicitation, STREME [31]. STREME identified enriched motif sequences relative to a control set of shuffled input sequences generated by STREME. We used the motif comparison tool Tomtom to search the motif database “vertebrates (in vivo and in silico)” to identify best matches to known motifs [32]. We used Find Individual Motif Occurrences tool FIMO to scan sequences sets of sequences to determine which sequences had matches to identified motifs [33].

### 2.6 Luciferase assay

De novo discovered multiple repeats of Creb3l1 binding motifs were synthesised by Sigma–Aldrich as two long complementary oligonucleotides (Supplemental Table 1) with the insertion of restriction site overhangs for annealing and cloning into compatible sites of minimal promoter plasmid pGL4.23 (Promega, E8411). Promoter fragments were amplified from rat genomic DNA and cloned into luciferase reporter construct pGL4.10 (Promega, E6651). Primers details are found in Supplemental Table 1. Luciferase assays were performed as described previously using Dual Luciferase Reporter Assay System (Promega, E1910) [21]. To transfect DNA into cells Lipofectamine LTX (Thermo Fisher Scientific, 15338100) was used on cells seeded in 24-well plates. Plasmid constructs pGL4 promoter/motifs (0.05 μg/well), constitutively active Creb3l1 (Creb3l1CA) or empty pcDNA3.1 (0.45 μg/well), and control renilla reporter pRL-CMV (1 ng/well, Promega, E2261). Samples were collected 48 h post transfection.

### 2.7 Virus production and validation

Creb3l1 and non-targeting short hairpin RNAs (shRNAs) were cloned into pGFP-A-shAAV (OriGene, TR30034) as previously described [20]. We generated adeno-associated viruses (AAVs) expressing GFP and Creb3l1CA directed by a 2 kb fragment of the rat *Avp* promoter. AAV particles (AAV1/2) were produced using a helper free packaging system (Cell Biolabs, VPK-402 and VPK-421) to titres of 6 × 10^12^ (AAV1/2-shRNA constructs) and 1 × 10^12^ (AAV1/2-AVPp-GFP and AAV1/2-AVPp-Creb3l1CA) genome copies per ml as described previously [20]. AAV1/2-OXTp-Venus and AAV1/2-AVPp-tdtomato were produced by Dr. Valery Grinevich's lab at the University of Heidelberg, Germany.

### 2.8 Stereotaxic injections of virus into the SON and PVN

For stereotaxic injections, rats were anaesthetised by intramuscular administration of a medetomidine (0.25 mg/kg, Domitor, Vetoquinol) and ketamine (50 mg/kg, Narketan, Vetoquinol) mix and placed in a stereotaxic frame in the flat skull position. Stereotaxic injections were performed as described previously [17]. After surgery, the incision was closed and atipamezole (0.15 mg/kg, Antisedan, Zoetis) was administered intramuscularly and post-surgery analgesia with buprenorphine (0.05 mg/kg, Buprevet, Virbac) subcutaneously. The success of viral injections was verified at the end of each study by visualisation of the fluorescent reporter and/or qRT-PCR.

### 2.9 Isolation of RNA from the SON and PVN

The processing of brains for punching was randomised by using the balls in a bag principle to prevent processing bias. A 1 mm punch (Fine Scientific Tools, 18035–01) was used to collect SON and PVN samples from 100 μm coronal slices sectioned in a cryostat set at −20 °C as previously reported [17]. Total RNA was extracted using a Directzol RNA MiniPrep extraction kit (Zymo Research).

### 2.10 RNA sequencing

Animals received an injection of control virus in one SON and Creb3l1 knockdown virus in the other (n = 5 animals). An alphanumeric system was applied for the labelling of RNA samples. This allowed for the blind-analysis of the samples sent to the internal facility and the subsequent data analysis. Samples (n = 5 SONs for each group) for RNA-seq went through rigorous quality control checks to assess purity and integrity (Agilent BioAnalyzer RNA TapeStation). The average RIN value was 8.5 (range 8.0–9.0) (Supplemental Table 2). Poly(A) enriched bulk RNA-sequencing libraries were constructed using Illumina TruSeq Stranded mRNA kits (Bristol Genomics Facility, University of Bristol). The libraries were assessed for their quality using a Qubit dsDNA High Sensitivity DNA Kit (Thermo Fisher Scientific) and Agilent 2100 Bioanalyzer (Agilent Technologies, Agilent High Sensitivity DNA Kit). Libraries were loaded onto lanes of an Illumina NextSeq flowcell and sequenced using 75 bp paired end (PE) runs (Bristol Genomics Facility, University of Bristol). Each sample generated >30 million PE reads. RNA-seq alignment and subsequent data analysis were all performed in house using our high-performance computer (Dell PowerEdgeR820 12 core equipped with 512 GB RAM and 12x1TB HDD). RNA-seq reads were processed using RTA and CASAVA from Illumina's suite of sequencing software. This produced a series of compressed FASTQ files per library. All raw reads were merged per library, and pre-processed for quality assessment, adaptor removal, quality trimming and size selection using the FastQC toolkit to generate quality plots for all read libraries [34]. We adopted a phred30 quality cutoff (99.9% base call accuracy). The pipeline accepts RNA-seq post-trimmed data as input, before ultimately producing output tables of differentially expressed transcripts. Paired end (2 × 75 bp) raw input data is initially aligned with STAR to the sixth iteration of the *Rattus norvegicus* reference genome (Rn6) [35]. FeatureCounts is used to generate read counts for each gene present in the Rn6 genome annotation [36]. Our pipeline then uses the DEseq2 (v1.28.1) package in R to call differentially expressed genes [37]. This allows us to predict differentially expressed genes with high confidence, and to utilise the predictions with low p-values in our downstream validation. Benjamini-Hochberg correction was used for multiple comparison. The analysis was sufficiently powered (n = 5) to reduce the false discovery rate, and to enable systems level analysis [38]. Differentially expressed genes with p adjusted values of <0.05 are considered significant.

Gene Ontology (GO) and Kyoto Encyclopaedia of Genes and Genomes (KEGG) pathway analyses were performed in ShinyGO v.66 [39]. GO and KEGG analysis for ChIP-seq target genes were performed by comparing with a background of all protein-coding genes in the rat genome. All expressed genes by RNA-seq were filtered for baseMean >10 and used as the background list. Overlap analyses of differentially expressed genes and ChIP-seq targets were performed using VENNY 2.1.0 [40]. Regularised log transformation was applied for principal component analysis. Heatmaps were generated from z score normalised gene expression data in GraphPad Prism version 9.

### 2.11 Real-time quantitative reverse transcription PCR

Total RNA concentrations were determined using a Nanodrop 2000 spectrophotometer (Thermo Fisher Scientific). cDNA synthesis and qRT-PCRs were carried as described previously [20]. For relative quantification of gene expression, the 2^−ΔΔCT^ method was employed [41]. The internal control gene used for these analyses was the housekeeping gene *Rpl19* [16,17,19]. The qRT-PCR primer sequences can be found in Supplemental Table 1.

### 2.12 Immunofluorescence

Perfusion fixation of tissue and slicing of brains was performed as described previously [17]. Slices were washed three times for 5 min each in PBS. Immunofluorescent staining was performed as described previously [20]. Details of the antibodies used in this study can be found in Supplemental Table 3. Images were captured using a Leica DMI6000 inverted epifluorescence microscope with Photometrics Prime 95 B sCMOS camera and Leica LAS-X acquisition software.

### 2.13 Statistical analysis

Image brightness and contrast adjustments were made to the whole image in Leica LAS X software. Statistical analyses and plotting of data were performed in GraphPad Prism version 9. Statistical differences between two experimental groups were evaluated using independent-sample unpaired t tests with Welch's correction. One-way ANOVA with Tukey's *post hoc* tests were used to determine the difference between more than two samples in in vitro cell studies with only a single influencing factor. Two-way ANOVA with a Holm-Šídák method *post hoc* test was used to compare control and experimental values at each timepoint in physiological studies with Creb3l1 knockdown under basal conditions. This Holm-Šídák method does not compute confidence intervals but offers greater statistical power to ensure that effects were not being missed. Two-way ANOVA with Fisher's LSD *post hoc* test was used for planned comparisons at day 1, 2, and 3 of WD where it was not sensible to have formal corrections for multiple comparisons in WD-induced protocol. Statistical analysis of qRT-PCR data was performed using the delta Ct values. A Grubbs' test was performed in GraphPad to identify any significant outliers with an Alpha = 0.05. Viral injection misses confirmed by qRT-PCR or through the visualisation of the GFP reporter expression were not included in analyses. The data are presented as the mean ± SEM where p ≤ 0.05 was considered statistically significant.

### 2.14 Data availability

We mined data from Creb3l1 ENCODE project ENCSR109YGM which was performed in human K562 cells by Dr Michael Snyder, Stamford (www.encodeproject.org/experiments/ENCSR109YGM/). We downloaded datasets from the ENCODE portal [42] using the following identifiers: ENCFF116CGU and ENCFF701TVD to visualise significant peaks in IGV 2.11.0. RNA-seq data from WD rat SON used for comparisons is available at the NCBI Gene Expression Omnibus GSE175461 (https://www.ncbi.nlm.nih.gov/geo/query/acc.cgi?acc=GSE175461). The datasets for ChIP-seq and RNA-seq have been banked in NCBI's Gene Expression Omnibus under SuperSeries GSE200402 (https://www.ncbi.nlm.nih.gov/geo/query/acc.cgi?acc=GSE200402). All novel materials and raw data are available to the community upon request to the corresponding author.

## 3 Results

### 3.1 ChIP-seq identification of the Creb3l1 target transcriptome

**Figure 1:**
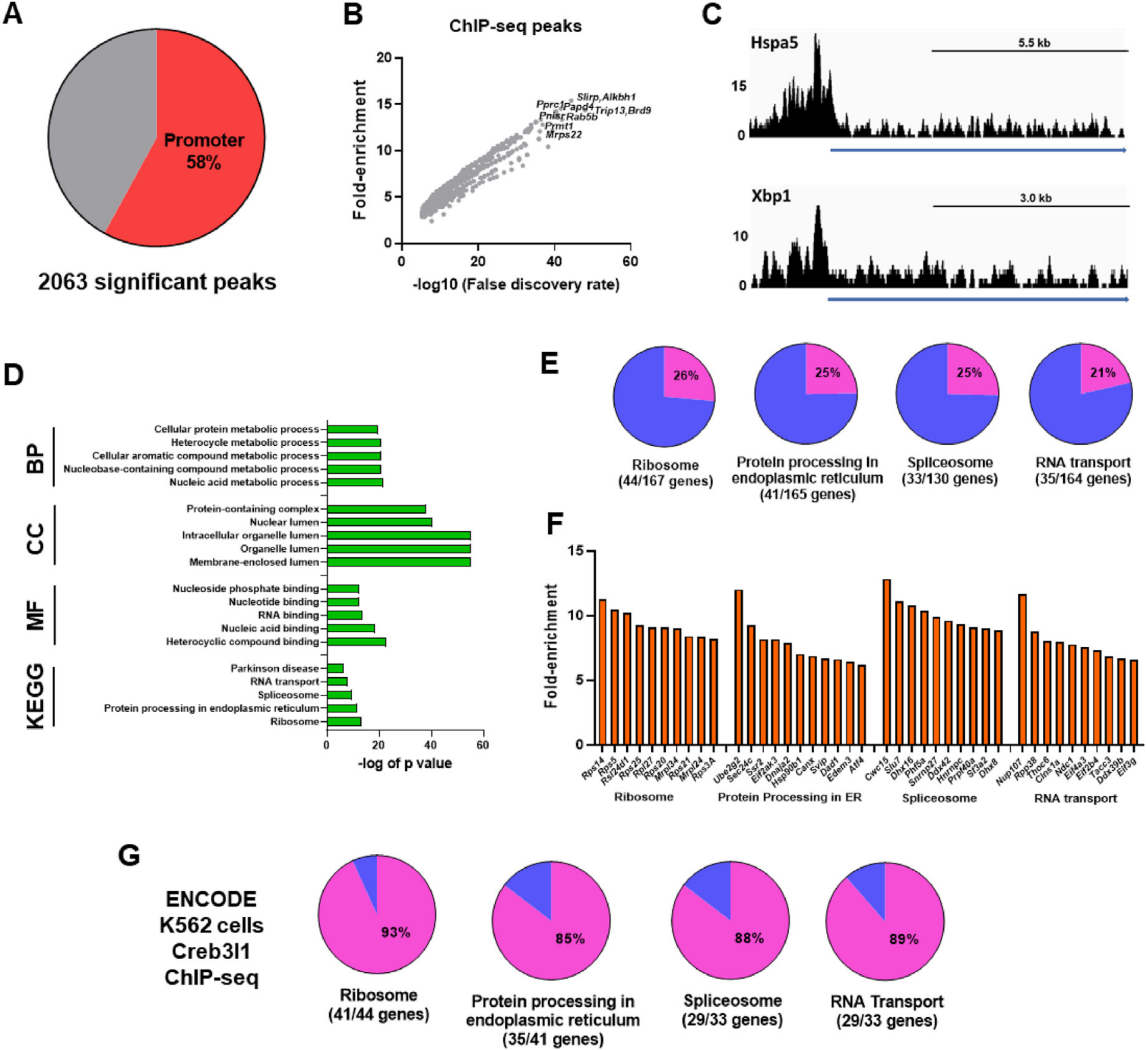
**ChIP-seq identification of the Creb3l1 target transcriptome in MCNs. A**, pie chart showing the percentage of peaks located in the promoter region. **B**, XY plot of the fold enrichment and false discovery for significant ChIP-seq peaks. **C**, ChIP-seq traces showing characteristic peaks for Creb3l1 close to the transcription start sites of *Hspa5* and *Xbp1*. The peak height is indicated, and the blue line indicates the gene. **D**, ChIP-seq GO analysis showing the top 5 most enriched terms for biological process (BP), cellular component (CC), molecular function (MF) and Kyoto Encyclopaedia of Genes and Genomes (KEGG) pathways. **E**, pie charts showing the percentage of Creb3l1 target genes for the top 4 enriched KEGG pathways. **F**, bar chart of the top 10 Creb3l1 target genes by fold-enrichment in the top 4 KEGG pathways. **G**, pie charts showing the percentage of overlapping genes between MCNs and K562 cell Creb3l1 ChIP-seq datasets.

We performed ChIP-seq to identify genome-wide binding sites for Creb3l1 in MCN enriched hypothalamus samples of WD rats. The analysis of ChIP-seq data identified 2063 significantly enriched peaks in the proximity of 1441 genes, with most peaks found to be in the promoter region (Figure 1A, Supplemental Table 4). The significant peaks were found to be enriched by 3-15-fold (Figure 1B). This included significant peaks in promoter regions of genes *Hspa5* which encodes the BIP protein and *Xbp1* (Figure 1C). To identify gene categories and pathways that might be regulated by Creb3l1 in MCNs, we performed pathway analysis using GO and KEGG (Figure 1D, Supplemental Table 5). We identified enriched terms in GO: Biological Process with metabolic processes involving nucleic acids comprising the most significantly enriched GO terms. We identified enriched terms in GO: Cellular Component with lumen, a membrane defined space within an organelle, such as ER, being a frequent term for the most significant GO terms. We identified enriched terms in GO: Molecular Function with the most significant terms representing nucleotide binding. The KEGG centred on neurodegenerative diseases and pathways that control core cellular processes. The data shows that Creb3l1 targets >20% of genes that compose the top 4 enriched KEGG pathways (Figure 1E). We show the top ten genes in each of these KEGG pathways by ChIP-seq fold-enrichment (Figure 1F). We next mined Creb3l1 ChIP-seq data from K562 cells in the ENCODE database. We found a considerable overlap of genome binding sites of Creb3l1 targets across both ChIP-seq datasets (Figure 1G).

### 3.2 De novo motif discovery of Creb3l1 DNA binding sites

**Figure 2:**
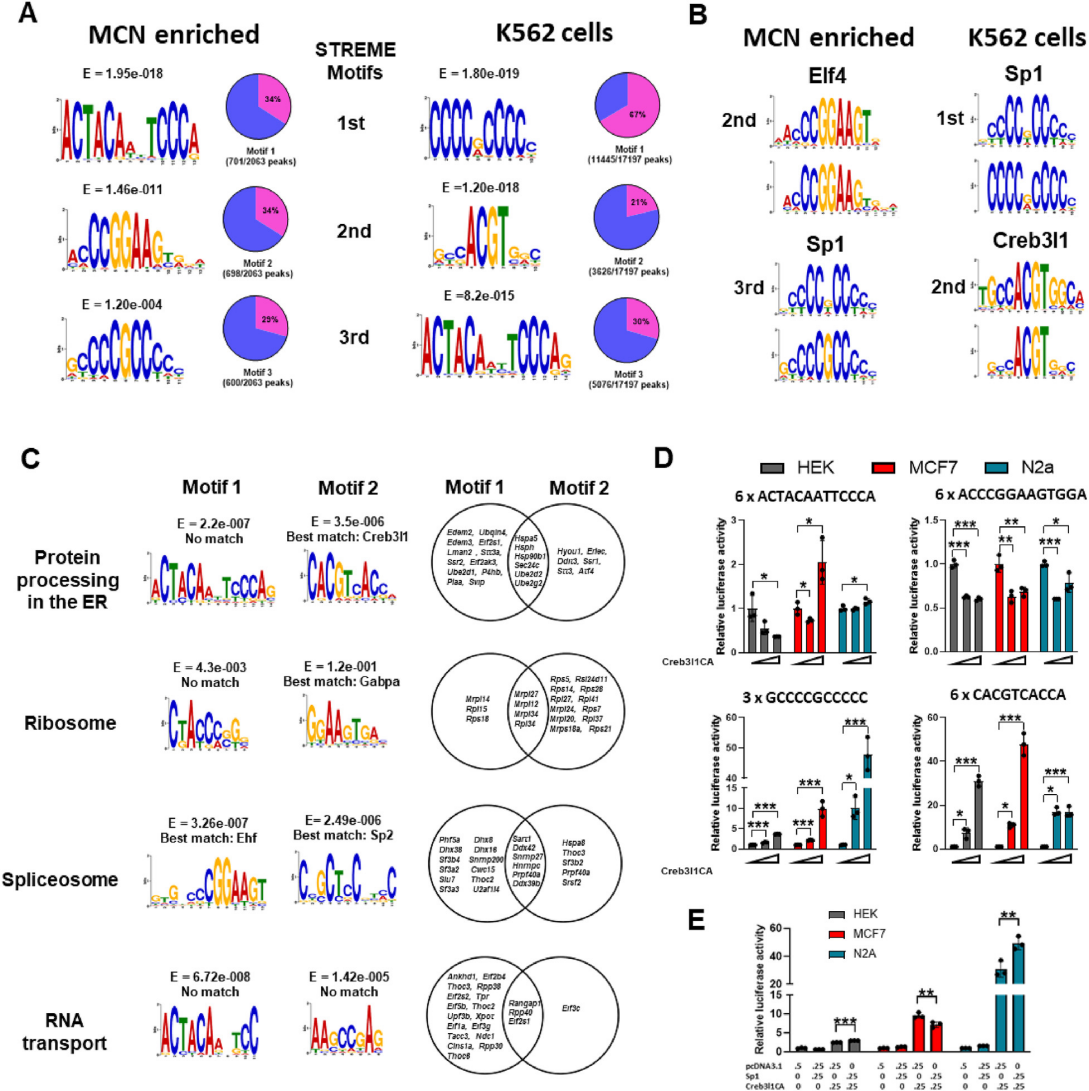
**De novo motif discovery of Creb3l1 DNA binding sites. A**, *de novo* motif analysis of Creb3l1 bound sites in MCNs and human K562 cells ChIP-seq datasets identified by STREME. The three most significant motifs by E value are shown. Pie charts show the percentage of significant peaks containing each discovered motif. **B**, motif comparisons were performed using Tomtom comparison tool. **C**, *de novo* motif discovery by STREME on peak sequences of genes in KEGG pathways Protein Processing in ER, Ribosome, Spliceosome, and RNA Transport. E values and Tomtom derived gene matches are indicated for each motif where present. Creb3l1 ChIP-seq target genes for each motif are shown in Venn diagrams. **D**, luciferase reporter vectors were constructed containing repeats of 4 discovered motifs. Assays were performed in HEK293T, MCF7 and N2a cells in the presence of low (0.05 μg) or high (0.45 μg) transfection concentrations of Creb3l1CA. **E**, luciferase assays for overexpression of Sp1 and Creb3l1CA with repeat plasmid GCCCCGCCCCC in HEK293T, MCF7 and N2a cells. Luciferase expression was normalised to the expression of the renilla luciferase control reporter vector and to luciferase expression in untreated cells for each cell-line. Values are means + SEM of n = 3 per group. ∗p ≤ 0.05, ∗∗p ≤ 0.01, ∗∗∗p ≤ 0.001.

We used the STREME motif discovery algorithm integrated in the MEME suite to identify ungapped enriched motifs in both MCN enriched and K562 cell ChIP-seq datasets. We report here the top three motifs calculated by STREME (Figure 2A). Two pairs of matching motifs were identified in the top 3 ranked motifs in MCN enriched and K562 datasets, and both represent novel sites for potential interaction by Creb3l1. The three most significant motifs were present in 29–34% of DNA peaks for the MCN enriched and 21–67% of DNA peaks for K562 cells. We performed motif comparisons using the Tomtom motif comparison tool (Figure 2B). There were no matches for ACTACATTCCCAR, whilst CCCCGCCCCC best matched that of a Sp1 TF binding sequence, ACCCGGAAGTGGA a Elf4 TF binding sequence, and GYCACGTGRC a Creb3l1 binding sequence.

We proposed that promiscuity of Creb3l1 binding, a feature of many TFs, could underlie distinct pathways or cellular processes being regulated. Therefore, we performed *de novo* motif discovery by STREME, but this time limited to peak sequences in KEGG pathways Protein Processing in ER, Ribosome, Spliceosome, and RNA Transport (Figure 2C). These data suggest that Creb3l1 DNA binding sequences may confer specificity to target pathways. Notably, the presence of a canonical Creb3l1 DNA binding motif in the pathway Protein Processing in ER is consistent with reports of Creb3l1 involvement in ER homeostasis [22,43]. We show Creb3l1 targets genes that possess each motif in overlapping Venn diagrams (Figure 2C).

We created luciferase reporter activity assays to investigate Creb3l1 actions on 4 identified motifs (Figure 2D). The overexpression of Creb3l1CA with repeat ACTACAATTCCCA significantly (one-way ANOVA) altered promoter activity in HEK293T (F_2,6_ = 8.96, p = 0.016), MCF7 (F_2,6_ = 16.30, p = 0.0038) and N2a (F_2,6_ = 9.44, p = 0.014) cells. Creb3l1CA decreased ACTACAATTCCCA promoter activity at high concentrations in HEK293T cells, decreased at low and increased at high concentrations in MCF7 cells, and increased activity at high concentrations in N2a cells.

The overexpression of Creb3l1CA with repeat ACCCGGAAGTGGA significantly (one-way ANOVA) altered promoter activity in HEK293T (F_2,6_ = 182, p < 0.0001), MCF7 (F_2,6_ = 22.66, p = 0.0016), and N2a (F_2,6_ = 28.94, p = 0.0008) cells. The overexpression of Creb3l1CA decreased ACCCGGAAGTGGA promoter activity at low and high concentrations in HEK293T, MCF7, and N2a cells. This suggests that DNA sequence ACCCGGAAGTGGA serves as a Creb3l1 repressor element in gene promoters. The overexpression of Creb3l1CA with GCCCCGCCCCC and CACGTCACCA significantly (one-way ANOVA) altered promoter activity in HEK293T (F_2,6_ = 738, p < 0.0001; F_2,6_ = 202, p < 0.0001), MCF7 (F_2,6_ = 61.0, p = 0.0001; F_2,6_ = 204, p < 0.0001) and N2a (F_2,6_ = 150, p < 0.0001; F_2,6_ = 86.70, p < 0.0001) cells. The overexpression of Creb3l1CA increased GCCCCGCCCCC and CACGTCACCA promoter activity at low and high concentrations in HEK293T, MCF7, and N2a cells. This suggests that DNA sequences GCCCCGCCCCC and CACGTCACCA serve as Creb3l1 enhancer elements in gene promoters. This agrees with studies showing that Creb3l1 binds to GC rich sequences and G-box like sequences to enhance gene expression [17,44].

The combinational binding of TFs to enhancers is known to result in synergistic activity that is essential for the shaping of gene expression. We investigated the possibility that Creb3l1 and Sp1 act together to form a common transcriptional output by binding to motif GCCCCGCCCCC (Figure 2E). The dual transfection of Creb3l1 and Sp1 with GCCCCGCCCCC luciferase construct resulted in an additive increase in luciferase activity by one-way ANOVA with Tukey's *post hoc* test in HEK293T (p < 0.001) and N2a (p = 0.002) cells. In contrast, we observed diminished luciferase activity in MCF7 cells (p = 0.0011). Studies have shown that Sp1 binds to several proteins which can differentially regulate Sp1-dependent transcription [45].

### 3.3 Identification of Creb3l1 as a regulator of the PERK pathway

**Figure 3:**
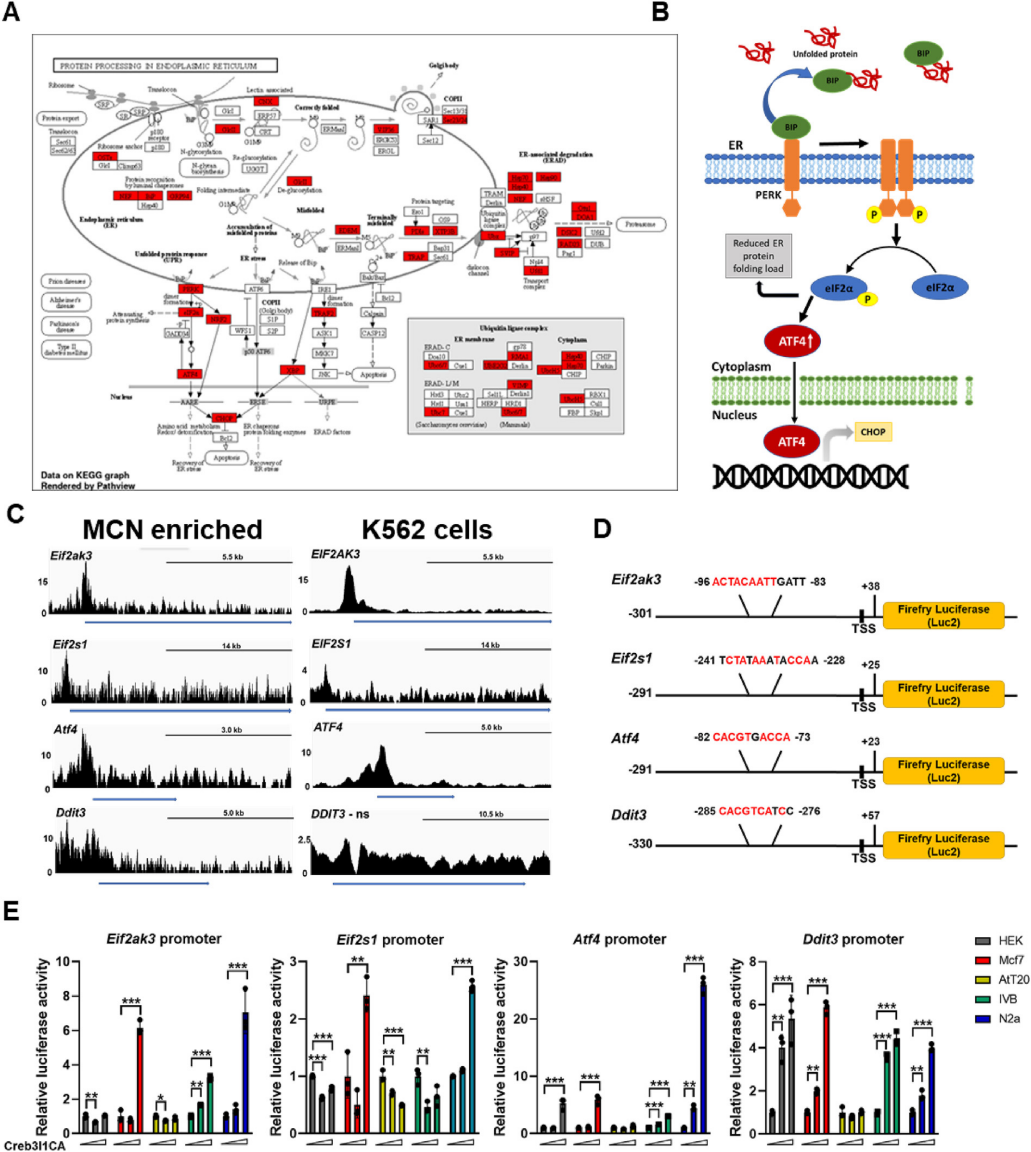
**Identification of Creb3l1 as a regulator of the PERK pathway. A**, integration, and visualisation of Creb3l1 ChIP-seq data in the KEGG pathway for Protein Processing in ER was generated by Pathview. The network components highlighted in red show Creb3l1 targets in this pathway. **B**, diagram of components of the PERK arm targeted by Creb3l1. **C**, ChIP-seq traces show significant peaks form Creb3l1 binding near to the transcription start sites of *Eif2ak3*, *Eif2s1*, *Atf4* and *Ddit3* in MCN enriched samples and *EIF2AK3*, *EIF2S1* and *ATF4* in human K562 cells. ns = not significant. The peak height is indicated on the left and the blue line indicates the gene. **D**, luciferase reporter vectors were constructed for the promotor regions of PERK pathway components *Eif2ak3*, *Eif2s1*, *Atf4* and *Ddit3* using the bound regions identified by ChIP-seq. The location and sequences of *de novo* discovered motifs in each promoter region are indicated. **E**, luciferase reporter assays were performed in HEK293T, MCF7, AtT20, 4 B and N2a cells in the presence of low (0.05 μg) or high (0.45 μg) transfection concentrations of Creb3l1CA. Luciferase expression was normalised to the expression of the renilla luciferase control reporter vector and to luciferase expression in control treated cells for each cell-line. Values are means + SEM of n = 3 per group. ns, not significant; ∗p ≤ 0.05, ∗∗p ≤ 0.01, ∗∗∗p ≤ 0.001.

As the ER is the gateway for proteins entering the secretory pathway, a pathway which neurones rely upon to secrete vast amounts of protein, we asked about Creb3l1 target genes that encode ER proteins. To do this we visualised the pathway Protein Processing in ER in Pathview (Figure 3A). This revealed ER target genes and processes essential to ER function including ER chaperones, UPR pathways, and ERAD. As mentioned earlier, Creb3l1 is a known to be a TF of ER master regulator BIP and UPR target XBP1. However, the data here supported a more comprehensive role for Creb3l1 in UPR pathway control, particularly genes in the PERK signalling arm (Figure 3B). Visualisation of ChIP-seq traces for genes *Eif2ak3* (encodes the protein PERK), *Eif2s1* (encodes the protein eIF2α), *Atf4* and *Ddit3* (encodes the protein CHOP) showed that Creb3l1 binds near to their transcription start sites (Figure 3C). We turned to Creb3l1 ChIP-seq data from K562 cells to strengthen these findings. In agreement, ChIP-seq traces showed that Creb3l1 binds near to the transcription start sites *EIF2AK3*, *EIF2S1*, and *ATF4*, but not *DDIT3*.

We next investigated genes in the PERK pathway as direct transcriptional targets of Creb3l1. We created luciferase promoter constructs for rat *Eif2ak3*, *Eif2s1*, *Atf4* and *Ddit3* (Figure 3D). We performed luciferase reporter assays in five different cell-lines to comprehensively describe the actions of Creb3l1 on each promoter (Figure 3E). There were significant (one-way ANOVA) alterations to *Eif2ak3* promoter activity in HEK293T (F_2,6_ = 25.36, p = 0.0012), MCF7 (F_2,6_ = 293, p < 0.0001), AtT20 (F_2,6_ = 7.50, p = 0.023), 4 B (F_2,6_ = 501, p < 0.0001), and N2a (F_2,6_ = 64.50, p < 0.0001) cells. Tukey *post hoc* analyses for *Eif2ak3* promoter assays showed that Creb3l1 significantly enhanced promoter activity in MCF7, 4 B, and AtT20 cells. In contrast, *Eif2ak3* promoter activity was repressed in HEK293T and AtT20 cells at the low transfection concentration. There were significant (one-way ANOVA) alterations to *Eif2s1* promoter activity in HEK293T (F_2,6_ = 111, p < 0.0001), MCF7 (F_2,6_ = 31.1, p = 0.0007), AtT20 (F_2,6_ = 54.17, p = 0.0001), 4 B (F_2,6_ = 11.6, p = 0.0087), and N2a (F_2,6_ = 701, p < 0.0001) cells. The *post hoc* analyses for *Eif2s1* promoter assays by Tukey showed that Creb3l1 can either enhance (MCF7, N2a) or repress (HEK, AtT20, 4 B) promoter activity in a cell-line dependent manner. There were significant (one-way ANOVA) alterations to *Atf4* promoter activity in HEK293T (F_2,6_ = 54.69, p = 0.0001), MCF7 (F_2,6_ = 115, p < 0.0001), AtT20 (F_2,6_ = 7.08, p = 0.026), 4 B (F_2,6_ = 1152, p < 0.0001) and N2a (F_2,6_ = 704, p < 0.0001) cells. The *post hoc* analyses for *Atf4* promoter assays by Tukey showed that Creb3l1 enhances promoter activity in all cell-lines apart from AtT20 cells. There were significant (one-way ANOVA) alterations to *Ddit3* promoter activity in HEK293T (F_2,6_ = 29.14, p = 0.0008), MCF7 (F_2,6_ = 534.3, p < 0.0001), 4 B (F_2,6_ = 225, p < 0.0001) and N2a (F_2,6_ = 244, p < 0.0001) cells. The *post hoc* analyses for *Ddit3* promoter assays by Tukey showed that Creb3l1 enhances *Ddit3* promoter activity in all cell-lines except for AtT20 cells. Taken together, these data support the PERK signalling pathway as a target of Creb3l1.

### 3.4 Integrative analysis of ChIP-seq and RNA-seq data reveals Creb3l1 as a key regulator of protein synthesis

**Figure 4:**
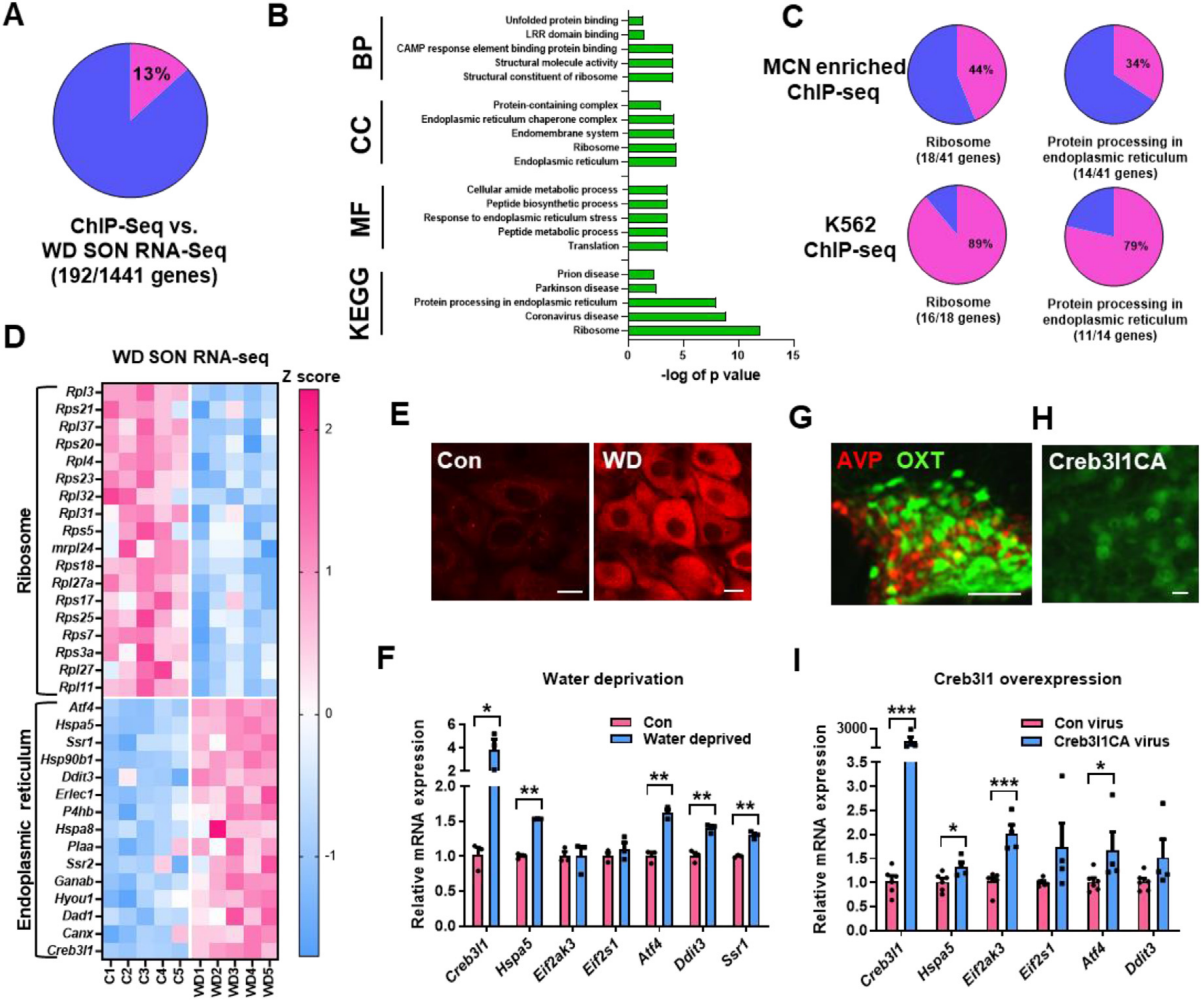
**Integrative analysis of ChIP-seq and RNA-seq data reveals Creb3l1 as a key regulator of protein synthesis. A**, pie chart displaying the percentage MCN Creb3l1 ChIP-seq target genes that are also differentially expressed in the SON WD RNA-seq data. **B**, GO and pathway analysis for overlapping genes (192) showing the five most enriched terms for biological process (BP), cellular component (CC), molecular function (MF) and Kyoto Encyclopaedia of Genes and Genomes (KEGG) pathways. **C**, gene overlap analyses of differentially expressed genes and Creb3l1 target genes in KEGG pathways, Protein Processing in ER and Ribosome. The percentage overlap is displayed in pie charts for MCN enriched and K562 cell ChIP-seq datasets. **D**, heat map of normalised SON WD RNA-seq data (C1-5, control; WD1-5, water deprived) for overlapping genes in KEGG pathways Ribosome and Protein Processing in ER. **E**, immunostaining of Creb3l1 expression in MCNs of control and WD SONs. **F**, relative mRNA expression was investigated by qRT-PCR in the SON of control compared to WD rats. **G**, adeno-associated virus constructs expressing AVPp-tdtomato (red) and OXTp-Venus (green) in the SON. **H**, immunostaining showed high levels of Creb3l1 expression in MCNs after injection of and AVPp-Creb3l1CA. **I**, relative mRNA expression by qRT-PCR in AVPp-GFP control virus injected and AVPp-Creb3l1CA overexpressing SONs. Values are means + SEM of n = 3–6 SONs per group. ∗p ≤ 0.05, ∗∗p ≤ 0.01, ∗∗∗p ≤ 0.001. Scale bars = 10 μm (E, I) and 100 μm (G).

We integrated SON RNA-seq and MCN enriched ChIP-seq data from WD to compare differentially expressed genes with target genes. We found 192 differentially expressed target genes by overlap analysis representing 13% of ChIP-seq targets (Figure 4A, Supplemental Table 6). We performed pathway analysis on this subset of Creb3l1 targets by GO and KEGG (Figure 4B, Supplemental Table 7). We identified enriched terms in GO: Biological Process; GO: Structural Constituent of Ribosome and GO: Unfolded Protein Binding. We identified enriched terms in GO: Cellular component; GO: Endoplasmic Reticulum and GO: Ribosome. We identified enriched terms in GO: Molecular Function; GO: Translation and GO: Response to Endoplasmic Reticulum Stress. The enriched pathways identified by KEGG again centred on neurodegenerative diseases and Ribosome and Protein Processing in ER. We compared SON differentially expressed genes and Creb3l1 ChIP-seq target genes in the pathways Ribosome and Protein Processing in ER (Figure 4C). The data showed that 44% of genes in the pathway Ribosome and 34% of genes in the pathway Protein Processing in ER were differentially expressed in the WD SON. Of these differentially expressed genes, 89% of ribosomal genes and 79% of ER genes were conserved Creb3l1 targets K562 cells. We constructed a heatmap to visualise the direction of differential gene expression for these two KEGG pathways (Figure 4D). We strikingly show that Creb3l1 ribosomal gene targets are decreased in expression, and in complete contrast, ER gene targets are increased in expression in WD RNA-seq data. In WD, Creb3l1 protein expression is robustly increased in MCNs in the SON (Figure 4E). We validated a sub-set of differentially expressed genes by qRT-PCR focusing predominantly on genes crucial to PERK signalling (Figure 4F). We show significantly increased abundance by t test of Creb3l1 (t = 4.29, p = 0.029), *Hspa5* (t = 20.11, p = 0.0024), *Atf4* (t = 7.69, p = 0.004), *Ddit3* (t = 5.79, p = 0.01), and signal sequence receptor subunit 1 (*Ssr1*) (t = 9.73, p = 0.004) in an independent set of samples. Thus, the integration of omics datasets suggests that Creb3l1 targets a set of ribosomal genes to repress their expression, whilst in parallel, targeting a set of ER genes to enhance their expression in the WD SON.

We next injected AAVs expressing Creb3l1CA into SONs under the direction of the rat *Avp* promoter to relate pathway changes to cell populations in the SON. This region of the *Avp* promoter restricts expression of transgenes to AVP MCN populations [[46], [47], [48]] (Figure 4G). We found Creb3l1 predominantly in the nucleus of transduced MCNs (Figure 4H). In support, studies of Creb3l1 have reported a strong nuclear localisation of this active form [27]. By qRT-PCR, we show that overexpression of Creb3l1CA (t = 14.86, p = 0.0003) in AVP MCNs significantly increases (t test) the abundance of transcripts crucial to PERK signalling; *Hspa5* (t = 2.53, p = 0.037), *Eif2ak3* (t = 5.20, P = 0.0009), and *Atf4* (t = 2.40, p = 0.043) mRNA (Figure 4I). This data confirmed that Creb3l1 can specifically target the PERK signalling pathway in SON MCN populations.

### 3.5 Integrative analysis of ChIP-seq data with Creb3l1 knockdown RNA-seq data

**Figure 5:**
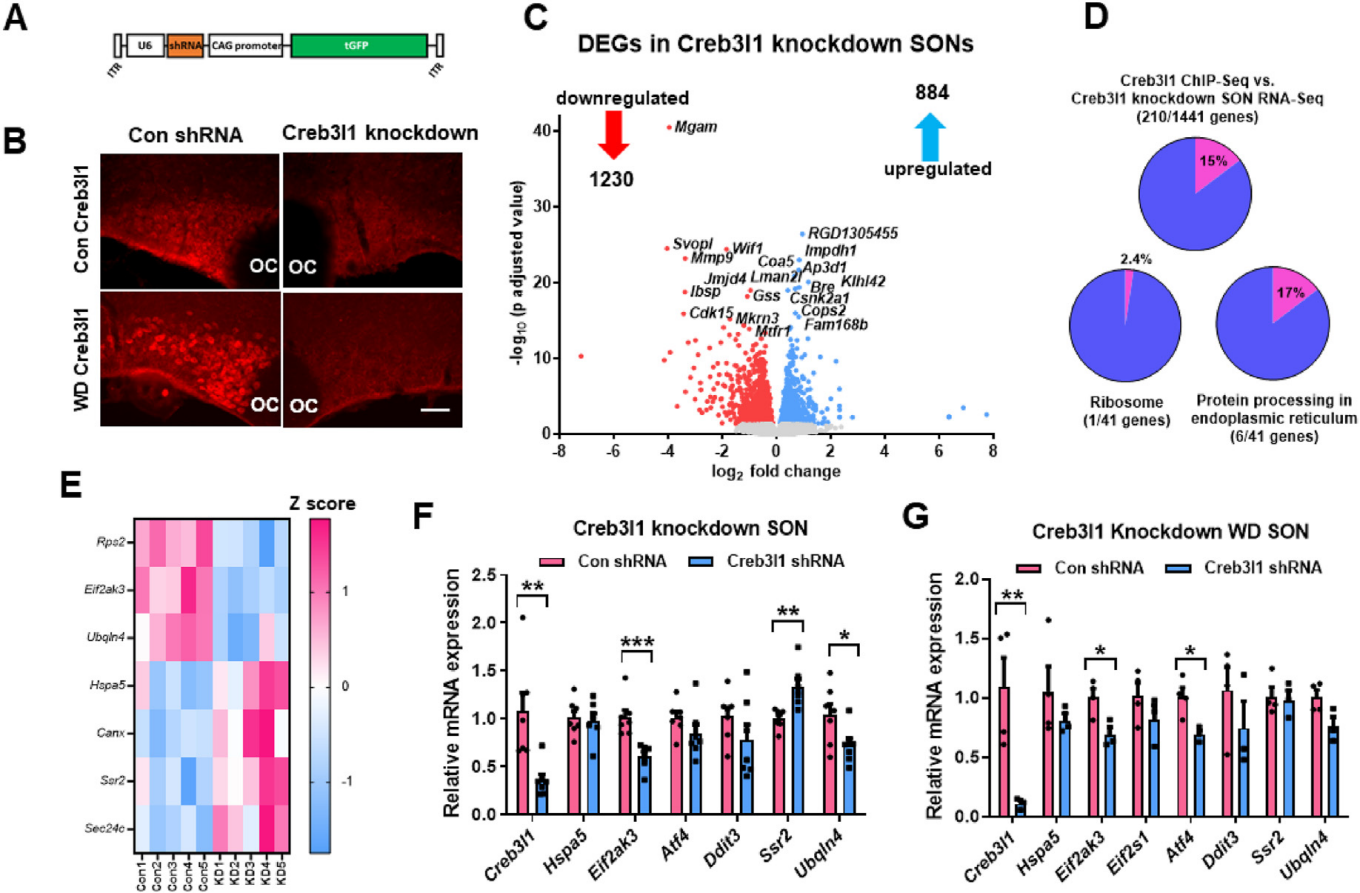
**Identification of Creb3l1 as a constitutive transcriptional regulator of PERK gene expression. A**, schematic of the construct used for viral-mediated gene knockdown. **B**, immunostaining of Creb3l1 in control and knockdown SONs in the basal state and in response to WD. **C**, volcano plot showing differentially expressed genes (DEGs) in Creb3l1 knockdown SONs. DEGs are displayed as red (downregulated) and blue (upregulated) dots. The top 20 most significant genes by p adjusted values are displayed. **D**, overlap analysis to compare differentially expressed genes by Creb3l1 knockdown SON RNA-seq and Creb3l1 target genes from MCN enriched ChIP-seq. Pie charts show the percentage of overlapping genes between the datasets and in KEGG pathways Protein Processing in ER and Ribosome. **E**, heat map for normalised SON Creb3l1 knockdown RNA-seq data of individual samples (Con 1–5, control; KD 1–5, knockdown) on overlapping genes in the KEGG pathways Ribosome and Protein Processing in ER. Relative mRNA expression was investigated by qRT-PCR in the SON of control (**F**) and WD (**G**) Creb3l1 knockdown animals. Values are means + SEM of n = 3–7 SONs per group. OC, optic chiasm. ∗p ≤ 0.05, ∗∗p ≤ 0.01, ∗∗∗p ≤ 0.001. Scale bars = 50 μm.

We next investigated transcriptome changes in SONs of Creb3l1 knockdown animals (Figure 5). To knockdown the expression of Creb3l1 we used AAVs expressing a Creb3l1 specific shRNA (Figure 5A). In a previous study, we validated this shRNA and this approach [20] here confirmed by immunostaining of Creb3l1 protein in SONs of control and WD animals (Figure 5B). Principal component analysis of bulk RNA-seq data revealed separation between the transcriptomes of control and Creb3l1 knockdown SONs (Supplemental Fig. 1). Differential expression analysis using DEseq2 identified a total of 2118 differentially expressed genes, 1230 of which were downregulated, with 884 being upregulated (Supplemental Table 8). Notably, the majority of genes 14230 did not pass the statistical significance threshold (p adjusted value ≤ 0.05). We show a volcano plot to display differentially expressed genes following Creb3l1 knockdown (Figure 5C). Displayed are the top 20 significant genes. We performed a simple overlap analysis to compare differentially expressed genes from Creb3l1 knockdown and target genes derived by ChIP-seq. We found 210 differentially expressed Creb3l1 target genes representing 15% of total ChIP-seq targets (Figure 5D, Supplemental Table 6). KEGG pathway analysis on this sub-set of genes did not reveal any enriched terms. However, we proceeded to analyse the overlap between Creb3l1 target genes from ChIP-seq with differentially expressed genes from Creb3l1 knockdown in the pathways Protein Processing in ER and Ribosome. This produced an overlap of 17% with Protein Processing in ER, but only a 1% overlap with Ribosome. We constructed a heatmap to visualise the direction of change of differentially expressed Creb3l1 targets (Figure 5E). In a separately derived set of samples, we performed qRT-PCR to validate these findings (Figure 5F). We confirmed changes to Creb3l1 (t = 3.623, p = 0.008), *Eif2ak3* (t = 4.617, p = 0.0007), signal sequence receptor subunit 2 (*Ssr2*) (t = 3.972, p = 0.002), and ubiquilin 4 (*Ubqln4*) (t = 2.30, p = 0.041), but not *Hspa5*. When SONs were stimulated by WD, we further demonstrate diminished expression of Creb3l1 (t = 6.097, p = 0.003), *Eif2ak3* (t = 3.26, p = 0.029) and *Atf4* (t = 3.90, p = 0.012) in response to Creb3l1 RNA knockdown (Figure 5G). Taken together, these data suggest that Creb3l1 is a constitutive enhancer to upgrade *Eif2ak3* expression in the SON.

### 3.6 Identification of Creb3l1 as a crucial enhancer of neuropeptide synthesis

**Figure 6:**
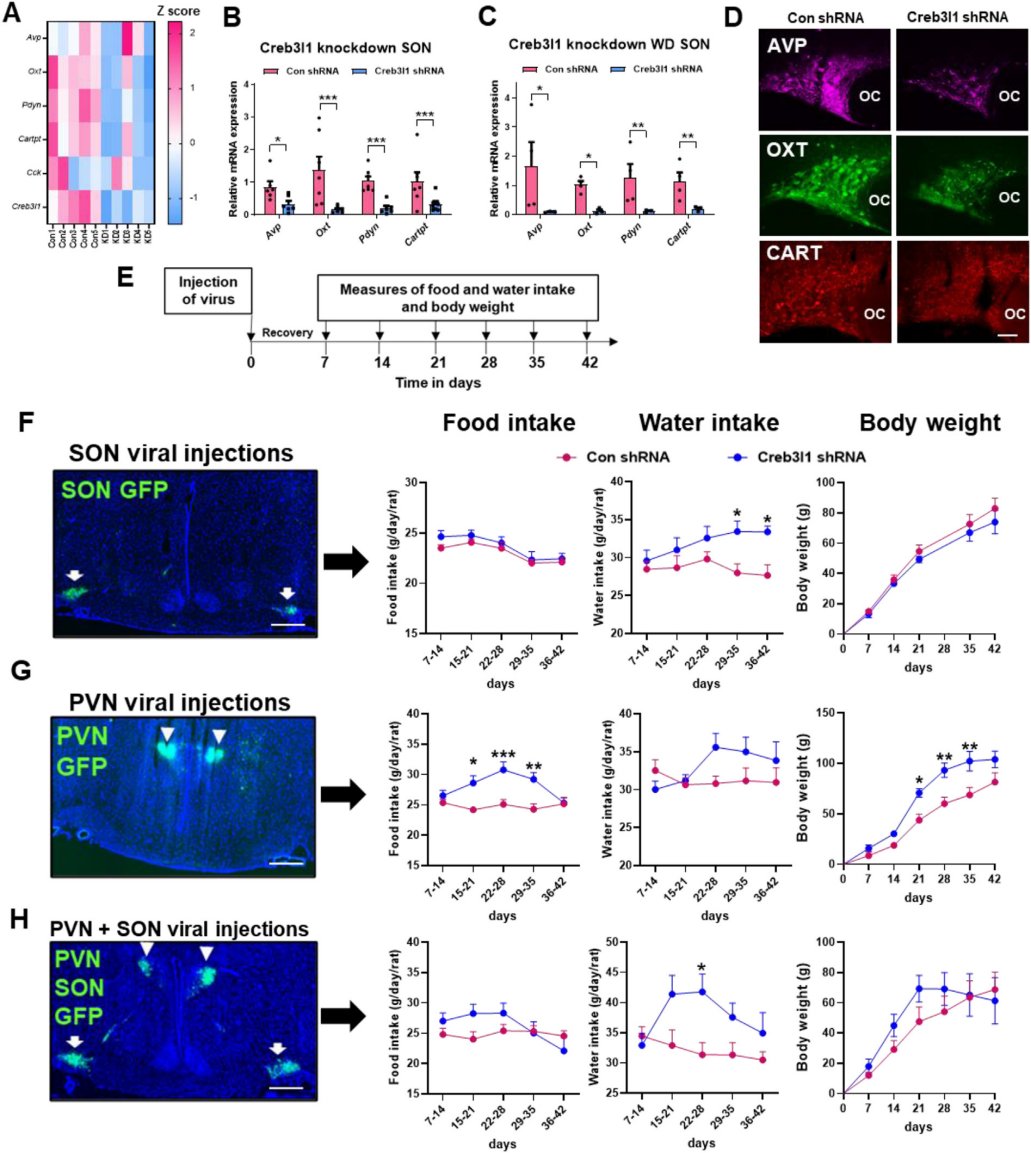
**Creb3l1 controls the major neuropeptide hormone outputs of the hypothalamo-neurohypophysial system. A**, heat map of normalised data for neuropeptide encoding genes in individual samples from control and Creb3l1 knockdown SONs. Relative mRNA expression was investigated by qRT-PCR in the SON of control (**B**) and WD (**C**) Creb3l1 knockdown rats. **D**, immunostaining of AVP, OXT and CART in control and Creb3l1 knockdown SONs. **E**, outline of the experimental protocol. Animals were injected with control or Creb3l1 knockdown virus bilaterally in the SON (**F**), PVN (**G**), or both PVN + SON (**H**). The success of viral injections was confirmed by GFP fluorescence in cryostat cut frozen sections. Arrow heads indicate GFP expression in the PVN and arrows indicate GFP expression in the SON. In this series of experiments food and water consumption and body weight were measured weekly by weight for a period of 6 weeks following virus delivery. Values are means + SEM of n = 4–6 animals per group. OC, optic chiasm. ∗p ≤ 0.05, ∗∗p ≤ 0.01, ∗∗∗p ≤ 0.001. Scale bars = 50 μm (D) and 500 μm (F–H).

We had the opportunity to investigate neuropeptide hormone synthesis in the SON in its entirety being informed by Creb3l1 knockdown RNA-seq data. We present a heatmap to visualise neuropeptide encoding gene expression in individual RNA-seq samples in control and Creb3l1 knockdown conditions (Figure 6A). Overall, these data pointed towards reduced expression of genes encoding neuropeptide hormones. In an independent set of samples, we validated changes to the expression of *Avp* (t = 2.74, p = 0.03), *Oxt* (t = 4.85, p = 0.0007), prodynorphin (*Pdyn*) (t = 5.34, p = 0.0008), and cocaine and amphetamine regulated transcript (*Cartpt*) which encodes the CART protein (t = 4.44, p = 0.0009) by qRT-PCR (Figure 6B). Note that an outlier was removed from *Avp* by Grubbs’ test. This effect on neuropeptide gene expression was also found in the PVN in the basal condition (Supplemental Fig. 2). In addition, we show decreased expression of *Avp* (t = 3.81, p = 0.028), *Oxt* (t = 4.34, p = 0.04), *Pdyn* (t = 5.42, p = 0.003), and *Cartpt* (t = 4.10, p = 0.01) in WD Creb3l1 knockdown SONs (Figure 6C). To address if gene expression changes translated to altered protein expression, we performed immunofluorescent staining for AVP-neurophysin II, OXT-neurophysin I and CART. We observed diminished expression of all three neuropeptide hormone precursors compared to control (Figure 6D).

### 3.7 Crucial physiological role for Creb3l1 in the hypothalamus

We next performed a series of physiological studies in Creb3l1 knockdown animals to establish the importance of Creb3l1 to SON and PVN neuroendocrine outputs and, by the very nature of these outputs, physiological functions. Our experimental protocol is summarised in Figure 6E. We measured food and water intake and body weight for 6 weeks after viral injection. Three experimental series were performed which included bilateral knockdowns of Creb3l1 in the SON (Figure 6F), PVN (Figure 6G) and PVN + SON (Figure 6H). Two-way ANOVAs were performed to determine if viral treatment (control vs. Creb3l1 shRNA) and length of treatment (time) had a significant effect on food, water intake, or body weight. A two-way ANOVA revealed that Creb3l1 knockdown (viral treatment) in the SON significantly altered water intake (F_1,45_ = 17.53, p = 0.0001), but had no effect on food intake or body weight (Figure 6F). There was no significant effect of time or interaction between time and virus treatment. Further, Holm-Sidak's multiple comparison test found that Creb3l1 knockdown animals drank significantly more water 28 days after viral treatment (29–35, p = 0.021; 36–42, p = 0.017) compared to controls. When Creb3l1 was knocked down in the PVN, a two-way ANOVA revealed significantly altered food intake (F_1,34_ = 33.16, p < 0.0001) and body weight (F_1,42_ = 36.99, p < 0.0001), but not water intake (Figure 6G). There was no significant effect of time on food intake, but a significant interaction between time and virus treatment was found (F_4,34_ = 3.84, p = 0.01). Holm-Sidak's multiple comparisons found that Creb3l1 knockdown in the PVN significantly increased food intake from 15 days after treatment compared to control (15–21 days, p = 0.008; 22–28, p = 0.0003; 29–35, p = 0.002) before returning to control measures. There was a significant effect of time (F_5,42_ = 54.42, p < 0.0001) on body weight, but no interaction between time and virus treatment. Holm-Sidak's multiple comparisons showed that PVN Creb3l1 knockdown animals significantly increased in weight from 21 days after treatment compared to controls (21, p = 0.019; 28 days, p = 0.004; 35, p = 0.004) consistent with altered food intake. The return towards control food intake and body weight measures by the end of this study suggests compensation for the loss of Creb3l1 in these animals. Dual knockdown of Creb3l1 in the PVN and SON significantly altered water intake (F_1,40_ = 13.42, p = 0.0007), but virus treatment had no significant effect on food intake or body weight (Figure 6H). There was no significant effect of time or interaction between time and virus treatment on water intake. Multiple comparisons performed by Holm-Sidak showed that knockdown animals drank significantly more water between 22 and 28 days compared to controls (p = 0.020). Thus, we establish the Creb3l1 target transcriptome as a key regulatory element for controlling neuropeptide biosynthesis and, as a result, define this TF as a crucial regulator of metabolic homeostasis.

### 3.8 A blunted response to WD following Creb3l1 knockdown in the SON and PVN

**Figure 7:**
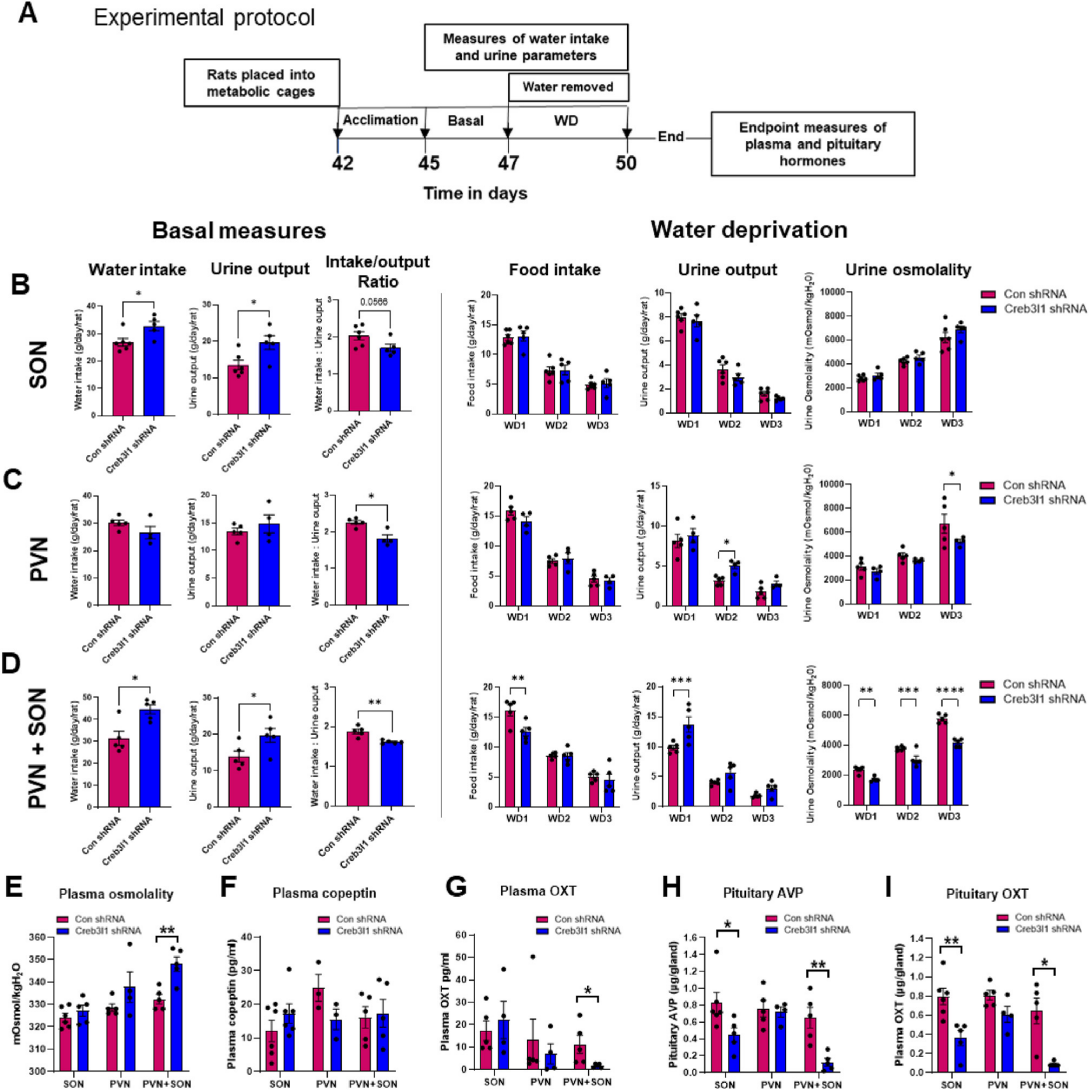
**Metabolic analyses reveal a crucial role for Creb3l1 in the physiological response to WD. A**, outline of the experimental protocol for Creb3l1 knockdown animals placed into metabolic cages. **B-D** measures of water intake, urine output, and water intake: urine output ratio in the hydrated state in SON (**B**), PVN (**C**), or both PVN + SON (**D**) knockdown animals. Water was then removed (WD) and food intake and urine parameters, volume, and osmolality, were recorded daily. End of study measures of plasma osmolality (**E**), plasma copeptin (**F**), plasma OXT (**G**) and pituitary stores of AVP (**H**), and pituitary stores of OXT (**I**) were performed. Values are means + SEM of n = 4–6 animals per group. ∗p ≤ 0.05, ∗∗p ≤ 0.01, ∗∗∗p ≤ 0.001.

We stimulated the system by WD in the same group of animals (Figure 7A). In the basal state daily food intake was not altered. SON knockdown (Figure 7B): Increased water intake in these animals (t = 2.66, p = 0.028) occurred in parallel with the excretion of larger volumes of urine (t = 2.65, p = 0.032). These data support increased water intake to compensate for renal loss of water, consistent with an altered, though not significant (t = 2.19, p = 0.057), water intake to urine output ratio used as an indicator of hydration status. When these animals were WD all output measures were preserved. PVN knockdown (Figure 7C): Water intake to urine output ratios were significantly reduced (t = 3.762, p = 0.017). This occurred without significant changes to water intake or urine excretion. When the PVN was stimulated by WD, analyses by two-way ANOVA revealed significant changes to urine excretory volume (F_1,21_ = 6.662, p = 0.017) and osmolality (F_1,21_ = 4.810, p = 0.040). Multiple comparisons by Fisher's LSD test found that urine volume was increased on day 2 of WD (p = 0.028) and urine concentration decreased by day 3 of WD (p = 0.022). Taken together, these parameters suggest a reduced capacity of the kidney to conserve water during WD perhaps the result of blunted AVP and/or OXT secretion. SON and PVN knockdown (Figure 7D): Water intake (t = 3.465, p = 0.010) and urine excretion volume (t = 2.345, p = 0.049) increased and water intake to urine output ratio decreased (t = 4.499, p = 0.007). A two-way ANOVA revealed significant changes to food intake (F_1,24_ = 6.318, p = 0.019) with a significant effect of time (F_2,24_ = 98.29, p < 0.0001) and interaction between time and treatment (F_2,24_ = 3.782, p = 0.037). Multiple comparisons by Fisher's LSD test found that food intake was significantly reduced on day 1 of WD (p = 0.0012). There was a significant effect on urine output (F_1,24_ = 15.55, p = 0.0006), but no interaction between time and treatment. Multiple comparisons by Fisher's LSD test showed that urine output was significantly increased on day 1 of WD (p = 0.0006). There was a significant effect of SON and PVN knockdown on urine osmolality (F_1,24_ = 84.55, p < 0.0001) with a significant interaction between time of WD and knockdown (F_2,24_ = 7.901, p = 0.0023). Multiple comparisons by Fisher's LSD test found significantly lower urine osmolality on days 1 (p = 0.002), 2 (p = 0.0006) and 3 (p < 0.0001) of WD.

We performed plasma electrolyte and hormone analysis on samples collected at the end of the WD protocol. Knockdown of Creb3l1 only in the PVN + SON group resulted in increased plasma osmolality (Figure 7E; t = 4.03, p = 0.005). We used copeptin as a surrogate to measure AVP secretion as both peptides are secreted in equal amounts [49]. We found no differences in plasma copeptin in all experimental series (Figure 7F). Note that only 3 samples were measured for the PVN. Plasma OXT (Figure 7G) was decreased only in knockdown PVN + SON animals (t = 2.37, p = 0.045). The abundance of peptides in the pituitary gland at the end of experimentation provides a good metric for changes in peptide synthesis/secretion [12]. Pituitary AVP and OXT stores were depleted in SON (AVP: t = 2.46, p = 0.039; OXT: t = 3.73, p = 0.0047) and SON + PVN (AVP: t = 4.01 p = 0.01; OXT: t = 4.19, p = 0.013) knockdown animals (Figure 7H–I). Taken together, these physiological data establish Creb3l1 as key component that defines neuroendocrine cell secretory capabilities in the hypothalamus.

## 4 Discussion

We performed ChIP-seq to provide genome-wide identification of regions occupied by Creb3l1 in hypothalamic neuroendocrine cells. By integrating ChIP-seq with multiple RNA-seq transcriptome datasets we provide a comprehensive level of understanding about Creb3l1 and its global transcriptional regulatory pathways across systems and cell-types. Foremost, these analyses centred on the ability of this TF to coordinate core cellular processes with established outcomes that are fundamental to neuronal cell function and ultimately survival. This has resulted in the identification of a novel mechanism enacted by Creb3l1 that integrate into the UPR pathway to control translation. Through a series of hypothalamic knockdowns, we show that expression of Creb3l1 controls neuropeptide synthesis in the hypothalamus and thus metabolic homeostasis by coordinating hypothalamo-neurohypophysial system (HNS) outflows at multiple levels.

The identification of binding motifs for TFs is important to understand how genetic variation by mutation within a motif may impact upon gene regulation. Indeed, mutations in TFs and TF-binding sites have been found to underlie many diseases in humans [50]. Importantly Using *de novo* motif discovery tools, we identified several TF-DNA binding motifs for Creb3l1. This promiscuity of Creb3l1 for different DNA motifs tentatively suggests that this feature can steer this TF actions towards the regulation of specific cellular process, in particular the expression of ribosomal proteins. In addition to its recognised cognate binding site [51], we validated three novel motifs as DNA targets for regulation by Creb3l1. Interestingly, studies have shown that, similar to Creb3l1 [[52], [53], [54]], the high expression of Sp1 is associated with poor prognosis in many cancers [45]. Thus, there is the possibility that common gene sets are being controlled by both TFs in cancer.

A multiomics approach of integrating ChIP-seq data with transcriptomic datasets narrowed our focus to Creb3l1 target genes in Ribosome and ER pathways. A decrease in expression of ribosomal gene targets of Creb3l1 is interesting given the knowledge that WD adapts MCN morphology to promote protein synthesis [13]. A knockdown study in human glioma cells by Vellanki et al. suggests that a Creb3l1 instructs a gene network to regulate expression of proteins to alter cell morphology [55]. There is evidence that ribosomes regulate translation of mRNAs, rather than simply being effectors of translation. These are termed ‘specialised ribosomes’ and refer to ribosomes for the selective translation of subsets of mRNAs that shape key events in gene regulation [56]. Indeed, studies have reported differences in the composition of ribosomal proteins in situations of cellular stress [57]. Therefore, it is possible that the altered ribosome landscape in 10.13039/100004696WD may promote genes that support demands for neuropeptide synthesis and secretion. These findings are complemented by early histological observations of stimulated MCNs which have increased numbers of nucleoli [13]. As the job of the nucleolus is to produce ribosomes these data agree with changes to ribosome biogenesis in this model.

In an earlier study we showed that WD leads to “physiological ER stress” in SON MCNs [16]. It is known that BIP is a transcriptional target of Creb3l1 in the ER stress response [27,55]. The importance of UPR control mechanisms to MCN survival has been demonstrated in mice where the selective knockdown of BIP in AVP neurones results in their progressive cell death [58]. We found that Creb3l1 regulates BIP expression in AVP MCNs and have demonstrated the potential of this TF to service a much larger network of genes essential for ER homeostasis. All omics comparisons converged on Creb3l1's ability to influence the PERK signalling cascade. In humans, loss-of-function mutations of PERK are the cause of Wolcott-Rallison Syndrome, which include severe insulin deficiency and permanent diabetes mellitus [59]. PERK knockout mice have severe defects in secretory cell types involved in bone formation and pancreatic β cells with impaired insulin trafficking and cell survival [60]. We found that Creb3l1 is necessary to sustain PERK gene expression by interactions with the PERK gene promoter. This is not the first link between Creb3l1 and PERK signalling. In a study of breast cancer, it was suggested that Creb3l1 was a target of downstream effector ATF4 [52]. We suggest that Creb3l1 upgrades the “classic” UPR pathway to allow for normally unprecedented levels of protein synthesis by specialised cell populations including neurones. More broadly speaking, dysregulation of the UPR pathway, including PERK signalling, is a major pathogenic mechanism in neurodegeneration and Creb3l1's involvement presents a new target for manipulation of this pathway.

We have previously reported that Creb3l1 regulates the synthesis of *Avp* and *Oxt* in the SON [[17], [18], [19], [20], [21]] and extend these findings here to include *Cartpt* and *Pdyn*. This global response has similarities with studies of Creb3l1 in other secretory cell-types where the synthesis of secretory proteins and cell secretory capacity are reported to be controlled by this TF [23,24,26,43]. Indeed, we also found that *Avp*, *Oxt*, *Cartpt*, and *Pdyn* were altered in expression in the Creb3l1 knockdown PVN, suggesting that Creb3l1 controls a gene regulatory network that regulates the expression of neuropeptides in PVN. To our knowledge only a single TF, single-minded 1 (Sim1), has received such acclaim in the hypothalamus. Sim1 heterozygous mice have significantly less AVP and OXT, as well as several other neuropeptides [61], which though has been contested [62], may not be the result of neuronal loss [63]. The expression of Sim1 is reduced in Creb3l1 knockdown RNA-seq data but further study is necessary to explain any relationship between these two TFs. Taken together, these data suggest that Creb3l1 drives the expression of secretory proteins and prepares the secretory pathway for increased protein load. This will help to mitigate problems with accumulation of misfolded proteins in the ER and maintain proteostasis to prevent cell death. Furthermore, Creb3l1 expression has also been described in additional neuroendocrine cell populations in hypothalamus including the suprachiasmatic nucleus and arcuate nucleus [17,64], suggesting that Creb3l1 may also regulate the expression of neuropeptide encoding genes in these nuclei. Thus, targeting of this TF may provide a new approach to regulate neuropeptide synthesis in neuronal cells.

The secretion of hormones from the HNS is crucial to maintain physiological homeostasis. The control of water intake via thirst and water output by regulating the release of AVP is crucial to maintain plasma osmolality. Animals with knockdown of Creb3l1 in the SON displayed classic signs of diabetes insipidus with increased fluid intake and the production of higher volumes of diluted urine (polyuria) compared to controls, likely due to insufficient release of AVP and OXT. We further identify a role for Creb3l1 in the PVN in the regulation of food consumption. The neuropeptide networks in the PVN are well-known to regulate food intake [65]. When administered directly into the PVN, AVP, OXT, and CART promote satiety [[66], [67], [68]]. Studies in mice have shown that activation of PVN AVP neurones by chemogenetic tools acutely inhibits food intake [69]. The literature regarding the role of PVN OXT neurones in food intake is far more expansive and at the same time conflicting [70]. The selective activation or inhibition of PVN OXT neurones has been shown not to alter feeding behaviour. However, the selective targeting of PVN OXT expression by lentiviral delivered shRNA was found to increase food intake in mice [71]. The apparent nature of Creb3l1 to universally control the synthesis and secretory mechanisms that govern neuropeptide release suggest that a collection of peptides may be responsible for the observed changes to ingestive behaviour. Thus, we establish Creb3l1 as a target for hypothalamic control of food intake.

## 5 Conclusions

In summary, we show that Creb3l1 creates a cellular environment for high levels of protein synthesis and secretion in neuroendocrine cells to meet physiological demands. One way this can be achieved is by direct modulation of the PERK signalling pathway to resist the accumulation of misfolded proteins to protect proteostasis. A second major process is through the regulation of ribosomal gene expression, a mechanism that may allow for the selective translation of subsets of mRNAs, specifically neuropeptide encoding genes in MCNs. The physiological data highlights Creb3l1 as a potential therapeutic target for hypothalamic control of food intake. With the recent advances in design strategies of targeting techniques this is now certainly becoming possible to target this TF [72]. The broad interest in these findings lies in Creb3l1's role is to control the UPR pathway foremost as Creb3l1 is being investigated as a target for cancer therapies [52]. This gene component may also explain the resilience of MCNs to neurodegenerative disease pathologies and ageing. The modulation of the PERK is one way of promoting neuroprotection and is a focus of new therapeutic strategies. Thus, we present Creb3l1 as new target for UPR modulation in disease.

## Author contributions

Conceptualization, MG., MPG., and DM.; Methodology, MG., MPG., AP., and DM.; Formal Analysis, MPG., BTG., and AP.; Investigation, MG., MPG., BTG., RF., and FA.; Writing – Original Draft, MPG; Writing – Review & Editing was performed by all authors; Funding Acquisition, DM., and VG.; Resources, FA. and VG; Data Curation, MPG., and BTG.
