# Supplementary material for: Transcription factor Creb3l1 maintains proteostasis in neuroendocrine cells
Source: Mol Metab. 2022 Jul 6;63:101542. doi: 10.1016/j.molmet.2022.101542 (PMC9294333; doi:10.1016/j.molmet.2022.101542)
Supplement: Multimedia component 1 [file mmc1.zip › Supplemental data/Supplemental Figures.docx]

**Supplemental Figures**


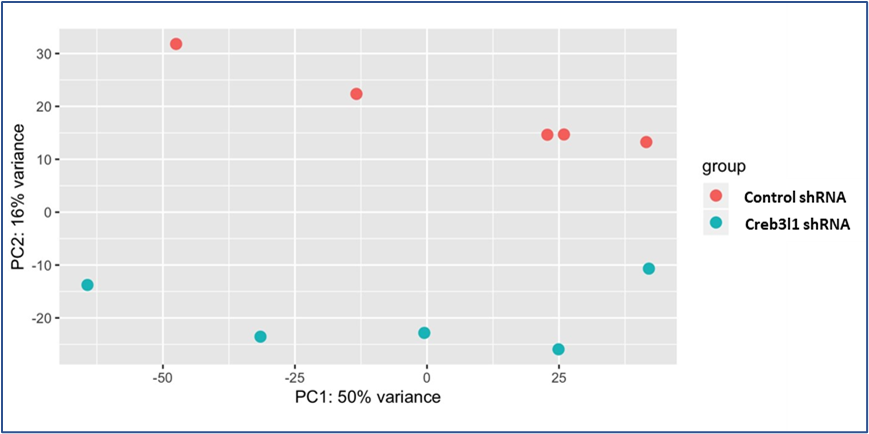


**Supplemental Fig. 1**. Principal component analysis showing distinct separation between control and Creb3l1 shRNA treated SONs.

**Supplemental Fig. 2**. **Knockdown of Creb3l1 in the PVN decreased the expression of neuropeptide hormone encoding genes.** Relative mRNA expression was investigated by qRT-PCR in the SON of euhydrated Creb3l1 knockdown rats. Animals were injected with control or knockdown virus unilaterally in the PVN. The success of viral injections was confirmed by GFP fluorescence in cryostat cut frozen sections. Knockdown of Creb3l1 significantly (t test) reduced Creb3l1 (t = 4.481, p = 0.007), *Avp* (t = 3.251, p = 0.02), *Pdyn* (t = 5.822, p = 0.007), and *Cartpt* (t = 5.197, p = 0.0013). Values are means +SEM of n = 4 - 5 animals per group. *p ≤ 0.05, **p ≤ 0.01, ***p ≤ 0.001.
