# Supplementary material for: Transcription factor Creb3l1 maintains proteostasis in neuroendocrine cells
Source: Mol Metab. 2022 Jul 6;63:101542. doi: 10.1016/j.molmet.2022.101542 (PMC9294333; doi:10.1016/j.molmet.2022.101542)
Supplement: Multimedia component 1 [file mmc1.zip › Supplemental data/Supplemental Table 1.docx]

| Gene | Forward primer | Reverse primer |
| --- | --- | --- |
| **qRT-PCR primers** | | |
| *Creb3l1* | GAGACCTGGCCAGAGGATAC | GTCAGTGAGCAAGAGAACGC |
| *Creb3l1* | GCCAACAGGACCCTGCTCCA | AGTGCCAGTCTGTGTGGCCG |
| *Hspa5* | TGGCACTATTGCTGGACTGA | CACATCGAAGGTTCCACCAC |
| *Eif2ak3* | CCTTAGCAAGCCAGAGGTGT | GGACTCCACAGTGAAAGGGA |
| *Eif2s1* | GGGAGTGGCTTGATTTGACC | ATGAAAACCGCAAGCCCAAT |
| *Atf4* | ATTCTTGCAGCCTCTTCCCT | TAAGCAGCAGAGTCAGGCTT |
| *Ddit3* | CTAGCTTGGCTGACTGAGGA | TGACTGGAATCTGGAGAGCG |
| *Ssr1* | CTCCGAGAAGGCTGCCC | CACAGACTGGACTGTTGCAC |
| *Ssr2* | TTAGCCGTCAGTCAAGCAGA | CTCCCTTCCACAGCGTATCT |
| *Ubqln4* | GTGAATACATGCAGGCTGGG | ACCAGTTGGAGCCATAACCA |
| *Avp* | TGCCTGCTACTTCCAGAACTGC | AGGGGAGACACTGTCTCAGCTC |
| *Oxt* | TGCCCCAGTCTTGCTTGCT | TCCAGGTCTAGCGCAGCCC |
| *Pdyn* | TGGATCGGCCATCCTATCAC | GCAGATCTCAAAGCCTGTGG |
| *Cartpt* | GCTGTGTTGCAGATTGAAGC | GGGACTTGGCCGTACTTCTT |
| *tGFP* | AACACCCGCATCGAGAAGTA | GTGCCCATCACCTTGAAGTC |
| *Gapdh* | ATGACTCTACCCACGGCAAG | CTGGAAGATGGTGATGGGTT |
| *Rpl19* | GCGTCTGCAGCCATGAGTA | TGGCATTGGCGATTTCGTTG |
| **Promoter primers** | | |
| *Eif2ak3_p* | CGGGGTACCAGGGGCATCCACTTACACCAA | CCGCTCGAGAGCCTTGTACAGACATCGCC |
| *Eif2s1_p* | CGGGGTACCGAAATAGAACATTACCTCTTCTTTAAGC | CCGCTCGAGTGAAGTGTATGTGTGAATCCCGA |
| *Atf4_p* | CGGGGTACCGACACCGGAAGTTCCGTAACGA | CCGCTCGAGCTACCACAGAGCAAGCAGAAATG |
| *Ddit3_p* | CGGGGTACCCGGTTGCCAAACATTGCATCATC | GGAAGATCTTCCGCCACCCGCTCATCTTCAAC |
| **Motif primers** | | |
| Motif 1 | CACTACAATTCCCAGACTACAATTCCCAGACTACAATTCCCAGACTACAATTCCCAGACTACAATTCCCAGACTACAATTCCCAG  TCGACTGGGAATTGTAGTCTGGGAATTGTAGTCTGGGAATTGTAGTCTGGGAATTGTAGTCTGGGAATTGTAGTCTGGGAATTGTAGTGGTAC | |
| Motif 2 | CACCCGGAAGTGGAACCCGGAAGTGGAACCCGGAAGTGGAACCCGGAAGTGGAACCCGGAAGTGGAACCCGGAAGTGGA  TCGATCCACTTCCGGGTTCCACTTCCGGGTTCCACTTCCGGGTTCCACTTCCGGGTTCCACTTCCGGGTTCCACTTCCGGGTGGTAC | |
| Motif 3 | CCACGTCACCACACGTCACCACACGTCACCACACGTCACCACACGTCACCACACGTCACCA  TCGATGGTGACGTGTGGTGACGTGTGGTGACGTGTGGTGACGTGTGGTGACGTGTGGTGACGTGGGTAC | |
| Motif 4 | CGCCCCGCCCCCGCTGCCAGATCCCATGGCCGTCATACTGGCCCCGCCCCCTTTCAGACACCCCATGCCCCGCCCCC  TCGAGGGGGCGGGGCATGGGGTGTCTGAAAGGGGGCGGGGCCAGTATGACGGCCATGGGATCTGGCAGCGGGGGCGGGGCGGTAC | |

**Primer table**

**Supplemental Table 1**. Oligonucleotides used in this study.
