# Supplementary material for: Transcription factor Creb3l1 maintains proteostasis in neuroendocrine cells
Source: Mol Metab. 2022 Jul 6;63:101542. doi: 10.1016/j.molmet.2022.101542 (PMC9294333; doi:10.1016/j.molmet.2022.101542)
Supplement: Multimedia component 1 [file mmc1.zip › Supplemental data/Supplemental Table 2.docx]

| **Sample name** | **RNA integrity number (RIN)** |
| --- | --- |
| Con1 | 8.2 |
| Con2 | 8.2 |
| Con3 | 8.5 |
| Con4 | 9 |
| Con5 | 8.8 |
| KD1 | 8.4 |
| KD2 | 8.7 |
| KD3 | 8.6 |
| KD4 | 9 |
| KD5 | 8 |

**Supplemental Table 7**. RIN values for Creb3l1 Knockdown SON RNA-seq samples.
