# Supplementary material for: Transcription factor Creb3l1 maintains proteostasis in neuroendocrine cells
Source: Mol Metab. 2022 Jul 6;63:101542. doi: 10.1016/j.molmet.2022.101542 (PMC9294333; doi:10.1016/j.molmet.2022.101542)
Supplement: Multimedia component 1 [file mmc1.zip › Supplemental data/Supplemental Table 3.docx]

|  | Antibodies | | | |
| --- | --- | --- | --- | --- |
| Antibody | | Dilution | Company | Cat No. |
| Rabbit polyclonal anti-CREB3L1 | | ChIP – 2 µg | Sigma-Aldrich | Cat No. HPA024069  RRID: AB_1854750 |
| Goat polyclonal anti-CREB3L1 | | 1:500 | R&D Systems | Cat No. AF4080  RRID: AB_2086044 |
| Goat polyclonal anti-CART | | 1:1000 | R&D Systems | Cat No. AF163  RRID: AB_2068569 |
| Mouse monoclonal anti-oxytocin-neurophysin, clone PS 38. | | 1:200 | Gifted by Professor Harold Gainer | Cat No. PS-38, RRID: AB_2315026 |
| Mouse monoclonal anti-vasopressin-neurophysin, clone PS 41. | | 1:200 | Gifted by Professor Harold Gainer | Cat No. PS41, RRID: AB_2313960 |
| Donkey anti-Rabbit IgG (H+L) Highly Cross-Adsorbed Secondary Antibody, Alexa Fluor™ Plus 488 | | 1:500 | Thermo Fisher Scientific | Cat No. A32790, RRID: AB_2762833 |
| Donkey anti-Goat IgG (H+L) Highly Cross-Adsorbed Secondary Antibody, Alexa Fluor™ Plus 594. | | 1:500 | Thermo Fisher Scientific | Cat No. A32758, RRID: AB_2762828 |
| Donkey anti-Rabbit IgG (H+L) Highly Cross-Adsorbed Secondary Antibody, Alexa Fluor™ Plus 594. | | 1:500 | Thermo Fisher Scientific | Cat No. A32754, RRID: AB_2762827 |
| Donkey anti-Mouse IgG (H+L) Highly Cross-Adsorbed Secondary Antibody, Alexa Fluor™ 647. | | 1:500 | Thermo Fisher Scientific | Cat No. A-31571, RRID: AB_162542 |

**Antibody table**

**Supplemental Table 3**. Details of antibodies used in this study.
